# Preserved anabolic skeletal muscle capacity in response to exercise and protein intake after critical illness

**DOI:** 10.1186/s13054-026-06418-7

**Published:** 2026-10-24

**Authors:** Arabella Fischer-Hammerschmied, Nicolas Tardif, Mirko Mandić, Thomas Gustafsson, William Apro, Johan Helleberg, Mariam Amouzandeh, Jonathan Grip, Dag Seldén, Olav Rooyackers, Martin Sundström Rehal

**Affiliations:** 1 Division of Anaesthesia and Intensive Care, Department of Clinical Science, Intervention and Technology (CLINTEC), Karolinska Institutet, Stockholm, Sweden; 2 Division of Cardiothoracic and Vascular Anaesthesia and Intensive Care Medicine, Department of Anaesthesia, Intensive Care and Pain Medicine, Medical University of Vienna, Vienna, Austria; 3 Department of Laboratory Medicine, Division of Clinical Physiology, Karolinska Institutet, Stockholm, Sweden; 4 Department of Clinical Physiology, Karolinska University Hospital, Stockholm, Sweden; 5 Department of Physiology, Nutrition and Biomechanics, Swedish School of Sport and Health Sciences, GIH, Stockholm, Sweden; 6 Department of Perioperative Medicine and Intensive Care (PMI), Karolinska University Hospital Huddinge, K32 Funktion PMI, Hälsovägen 13, Stockholm, 14186 Sweden

**Keywords:** Critical illness, Resistance training, Anabolism, Muscle protein synthesis, Rehabilitation

## Abstract

**Background:**

Low muscle mass and persistent weakness are common after critical illness, but the mechanisms behind impaired recovery are incompletely understood. The aim of this study was to investigate the skeletal muscle anabolic response to combined resistance exercise and protein intake in intensive care unit (ICU) survivors, compared to matched controls without a history of critical illness. We hypothesised that residual anabolic resistance limits muscle regeneration after ICU stay.

**Methods:**

Former patients were enrolled within 3 to 6 months of intensive care unit discharge. Muscle biopsies were obtained before and after resistance exercise using a flywheel leg extension ergometer, combined with ingestion of 24 g hydrolysed protein. Muscle protein fractional synthetic rate was determined from the incorporation of [ring-²H₅]phenylalanine into mixed-muscle protein during a primed continuous intravenous infusion. Anabolic and proteolytic pathways were measured using standard assays. Group-by-time differences were analysed using linear mixed models. The primary comparison was the between-group difference in pre-to-post change in muscle protein synthesis.

**Results:**

Twenty ICU survivors (60 ± 12 years, 30% female, BMI 26.9 (IQR 22.9–28.7), SAPS 3 62.3 ± 15.8) and 20 age- and sex-matched controls were included. ICU survivors had significantly lower quadriceps muscle thickness (*p* = 0.02) and handgrip strength (*p* = 0.04) than controls. In ICU survivors, muscle protein synthesis from plasma precursor amino acids was higher at baseline than in controls (0.041%/hour vs. 0.030%/hour, *p* = 0.009), but increased less (Δ 0.031 vs. Δ 0.040%/hour, *p* = 0.04). Post-intervention muscle protein synthesis was similar between groups (0.071%/hour vs. 0.070%/hour, *p* = 0.70). At baseline, ICU survivors showed lower activation of the autophagy-lysosomal pathway. In both groups, the intervention increased anabolic signalling, decreased initiation of autophagy and increased parts of the ubiquitin-proteasome pathway.

**Conclusions:**

In ICU survivors with persistent muscle loss and weakness, the increase in muscle protein synthesis after combined resistance exercise and protein intake was smaller than in controls, in the context of a higher baseline fractional synthetic rate. Post-intervention muscle protein synthesis rates were similar between groups. These findings provide the first mechanistic support for structured exercise-nutrition rehabilitation to promote muscle recovery after critical illness.

**Trial registration:**

This study was prospectively registered at ClinicalTrials.gov (NCT05261984), 2022-02-20.

**Graphical Abstract:**

**Figure:**
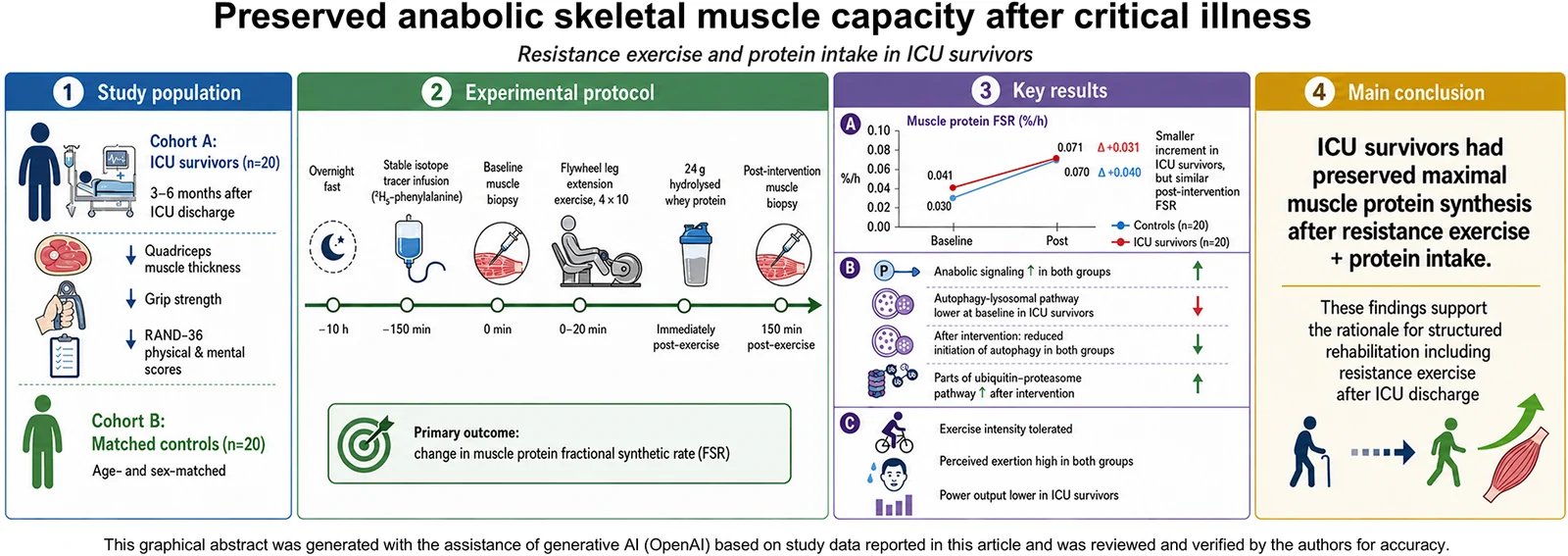

**Supplementary Information:**

The online version contains supplementary material available at https://doi.org/10.1186/s13054-026-06418-7.

## Background

Survivors of critical illness often experience sustained functional impairment after intensive care unit (ICU) discharge, including weakness, reduced physical performance, and shorter walking distance [1]. Physical disability is a major driver of morbidity in ICU survivors. It is associated with increased health care utilisation, work absence, loss of independence and reduced quality of life [2, 3].

Although ICU-acquired weakness (ICUAW) is multifactorial, the rapid and severe muscle wasting observed in critically ill patients is arguably the most prominent contributing cause [4]. A relative increase in proteolysis drives the acute muscle loss during ICU stay, with inflammation and immobilisation leading to a state of anabolic resistance and net negative muscle protein balance [5, 6].

While these acute changes are well described, the biological mechanisms governing muscle recovery after ICU discharge remain poorly defined. Few studies have investigated skeletal muscle structure and cellular function after ICU discharge. Emerging evidence suggests that recovery may be constrained by impaired regenerative capacity and persistent alterations in muscle gene expression [7, 8]. Consistent with this, functional recovery often plateaus despite structured rehabilitation, and post-ICU exercise interventions have shown only modest benefits [9]. Although these limited effects may partly reflect heterogeneity in exercise protocols and insufficient intensity, they also raise the possibility that critical illness induces durable changes in skeletal muscle homeostasis rather than transiently blunting anabolic responsiveness.

Understanding the mechanisms behind ICUAW was recently identified as a top priority in this field by an expert panel [10]. To date, no translational studies have investigated anabolic responsiveness of skeletal muscle in ICU survivors to stimuli such as resistance exercise or protein ingestion. To optimise recovery interventions, the physiological response to signals regulating skeletal muscle metabolism needs to be adequately characterised.

The aim of this study was to compare the anabolic response to a maximal anabolic stimulus with strenuous resistance exercise and concurrent protein ingestion in former ICU patients with that of matched controls. We hypothesised that residual anabolic resistance contributes to incomplete resolution of muscle wasting and weakness in this population.

## Methods

### Design, setting and participants

This prospective, single-centre, case-control non-randomised trial was conducted at Karolinska University Hospital. The first cohort included former adult patients admitted to one of the hospital’s intensive care units. Between March 2022 and November 2023, all admissions with an ICU length of stay of at least 72 h from the preceding three-month period were regularly screened. Former patients could be included within three to six months from ICU discharge. Major exclusion criteria were inability to consent or perform exercise, contraindications to biopsy, active malignancy, low illness severity during ICU stay and pregnancy. The second cohort of non-critically ill controls was recruited through public advertisements posted in the hospital area. ICU survivors and controls were matched according to sex and age within ± 5 years. Full criteria and screening procedures are provided in the supplement (Appendix S1, Table S1).

### Experimental protocol

**Fig. 1:**
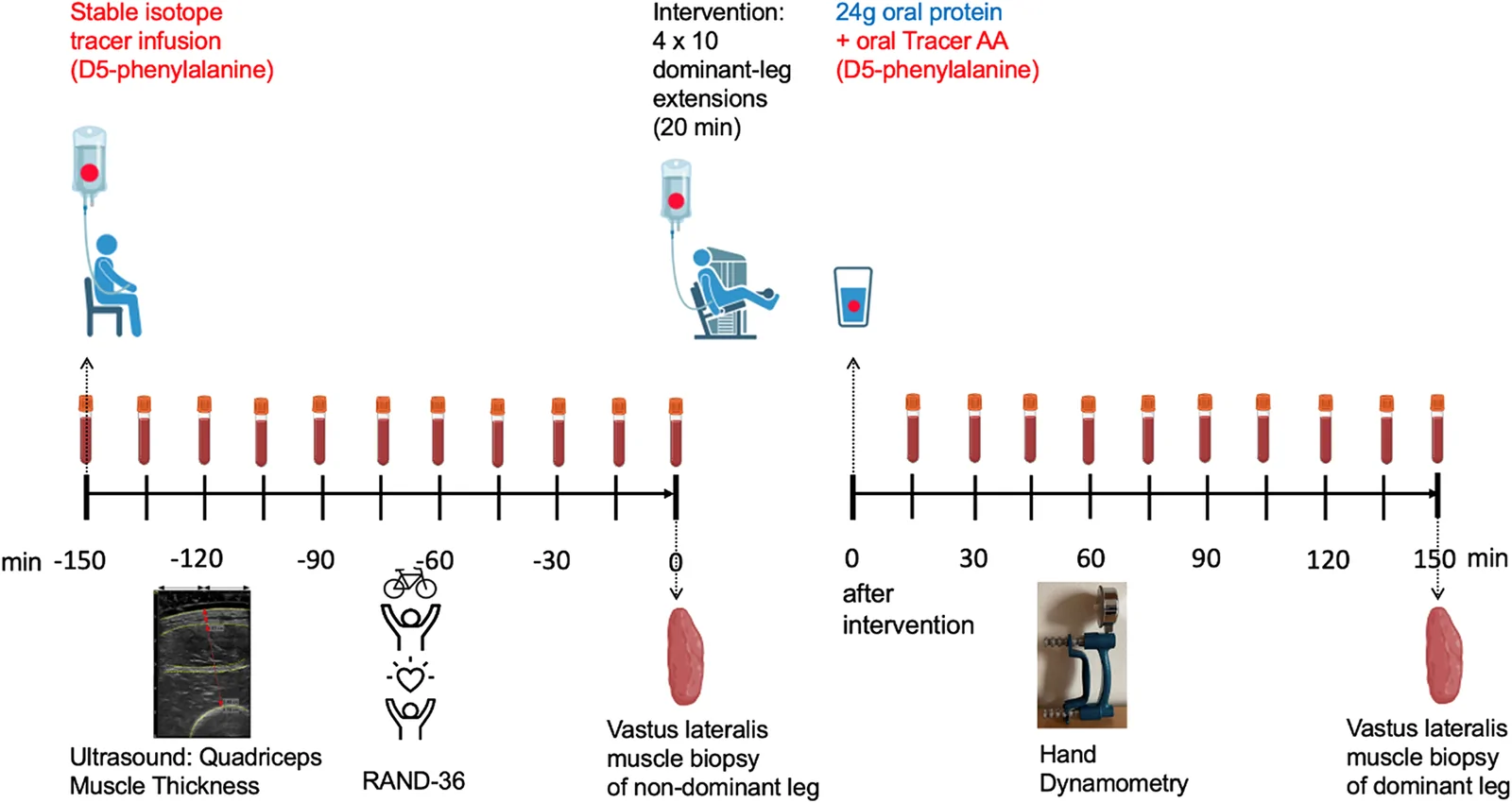
Experimental protocol. After an overnight fast, a primed intravenous infusion of ^2^H_5_-phenylalanine was started (time − 150 min) and arterial blood sampled every 15 min. A baseline muscle biopsy was taken from the non-dominant leg at time 0 (end of baseline period). Participants then performed 4 × 10 dominant-leg extensions, followed by immediate ingestion of 24 g of whey protein also enriched with ^2^H_5_-phenylalanine. A second biopsy was taken from the vastus lateralis muscle of the exercised leg 150 min after protein intake. Muscle protein fractional synthetic rates before and after the intervention were calculated from tracer enrichment in plasma, the intracellular precursor and in mixed muscle proteins. AA: amino acid, iv: intravenous. Created with elements of BioRender in PowerPoint

The protocol was identical in both cohorts (Fig. 1). After an overnight fast, participants received a primed continuous infusion of ²H₅-phenylalanine, with arterial blood sampled every 15 min for tracer enrichment and amino acid concentrations. After 150 min, a baseline vastus lateralis biopsy was obtained. Participants then performed four sets of 10 maximal coupled concentric-eccentric dominant-leg extensions on a flywheel ergometer after familiarisation and warm-up, and were actively coached by supervising staff to reach volitional fatigue within each set. Immediately after exercise, they consumed 24 g hydrolysed whey protein to augment the anabolic response. The drink was enriched with ²H₅-phenylalanine to maintain precursor enrichment. A second biopsy was obtained from the exercised leg 150 min after protein intake. Comprehensive descriptions of the tracer protocol, training equipment and exercise programme are available in the supplement (Appendix S2).

### Muscle mass, strength and health evaluations

Muscle thickness, strength and subjective health were assessed in ICU survivors and controls on the study day. Quadriceps muscle thickness was measured by ultrasound using a validated protocol [11], and muscle strength measured by hand dynamometry. Assessments of self-reported health included the functional comorbidity index (FCI), clinical frailty scale and the RAND-36 health survey, a 36-item self-reported measure of health-related quality of life across eight domains [12].

### Additional data collection

Clinical data from the hospitalisation period of former ICU patients and premorbid health conditions and functional status were extracted from the electronic medical records. This included admission diagnosis, length of ICU and hospital stay, illness severity on admission and daily SOFA scores, surgical status, organ support therapies and duration of mechanical ventilation, presence of sepsis, acute respiratory distress syndrome and acute kidney injury, body weight, and concurrent corticosteroid therapy.

### Outcomes

The primary outcome was the between-group difference in the change in skeletal muscle protein fractional synthetic rate (FSR), from baseline to post-intervention, estimated by the group-by-time interaction. To aid interpretation of this interaction, between-group differences in FSR at baseline and post-intervention were also estimated from the same model.

Secondary outcomes included pre- and post-intervention levels in signalling proteins for muscle protein anabolism and catabolism, and proteolytic activities related to the autophagy-lysosome and ubiquitin-proteasome pathways.

FSR was calculated using both plasma and intracellular muscle precursor enrichment. No hierarchy between these two calculations was prespecified. Comprehensive descriptions of the analytical techniques for all outcome measures are provided in the supplement (Appendix S3).

### Statistical analysis

Continuous baseline variables are described as mean ± SD or median (Q1-Q3), and compared using t-tests or Mann-Whitney U-tests as appropriate. Categorical baseline variables were compared using Pearson’s chi-squared test or Fisher’s exact test.

Primary and secondary outcomes were analysed using linear mixed models, with fixed effects for group, time point and group-time point interaction. Random effects were the participant and matching pairs of two participants. If the estimated variance of the random effect of the matching pair was zero, the term was removed and only the random effect of the participant ID was included in the linear mixed model. Post hoc simple-effect contrasts were calculated from the estimated marginal means of the linear mixed models to evaluate the change from baseline to post-intervention separately in controls and ICU survivors, and also the between-group differences at baseline and post-intervention. Results and corresponding graphs of primary and secondary outcomes indicate individual values and estimated marginal means (95% CI of estimated mean). Amino acid concentrations in plasma were plotted over time and analysed with an analogous linear mixed model without the random effect of the matching pair. No formal statistical testing was performed to assess steady-state conditions of isotopic enrichment in plasma.

A two-sided significance level of 0.05 was used. Analyses and graphs were performed using R version 4.3.3 and the lme4 package version 1.1–37 or Prism 11.0.0.

There are no prior publications in a post-ICU setting on which to estimate a treatment effect for the primary outcome. Initially a convenience sample of 40 ICU survivors and 20 matched controls was planned. After observing the actual rate of enrolment, the recruitment target was modified to 20 ICU survivors and 1:1-matched controls.

## Results

### Study cohorts

1491 patients with an ICU length of stay of at least 72 h were screened during the study period, with reasons for exclusion listed in Fig. S1. Participation took place between March 2022 and January 2024. 24 ICU survivors and 23 controls were enrolled, with 20 participants in each group completing the full protocol.

Demographic and clinical characteristics of ICU survivors are presented in Table 1. Mean age was 60 ± 12 years, and 30% of participants were female. Median time between ICU discharge and study day was 167 (153–176) days. Median length of ICU stay was 10 (9–14) days, and mean duration of invasive mechanical ventilation 9.1 ± 5.7 days (range 1–22 days). Mean SAPS 3 score was 62.3 ± 15.8 points. 19/20 patients were treated with vasopressors, and nearly one third received renal replacement therapy.

**Table 1:** Baseline characteristics and clinical data from ICU survivors

ICU survivors (*n* = 20)	
Female, no (%)	6 (30)
Age, years [min-max]	59.6 ± 12.4 [28.5–78]
Height, cm	176 ± 9
Weight, kg	81.5 (68.8–94.8)
BMI, kg.m^− 2^	26.9 (22.9–28.7)
Time between ICU discharge and study day, days [min-max]	167 (153–176) [119–185]
ICU length of stay, days [min-max]	10 (9–14) [4–29]
Hospital length of stay, days [min-max]	33 (24–55) [14–100]
Medical patient, no (%)	12 (60)
Surgical patient, no (%)	8 (40)
ICU Admission Diagnosis, no (%)	
Cardiac	6 (30)
Respiratory	5 (25)
Gastrointestinal	1 (5)
Neurological	4 (20)
Multiple organ failure	1 (5)
Systemic infection	3 (15)
ICU readmission, no (%)	2 (10)
Underwent surgery, no (%)	
Elective	2 (10)
Emergency	8 (40)
None	10 (50)
Type of surgery, no (%)	
Gastrointestinal surgery	2 (10)
Urologic surgery	1 (5)
Orthopaedic surgery	1 (5)
Neurosurgery	2 (10)
Cardiothoracic surgery	3 (15)
Soft-tissue wound surgery	1 (5)
Surgery day - ICU admission day, days [min-max]	0 (0-0.8) [-9 to 1]
Clinical Frailty Scale (CFS) before ICU stay, points, scale: 1–9	2 (2–3)
SAPS 3 score, points	62.3 ± 15.8
SOFA score at ICU admission, points	7.5 (5–10)
Sepsis during ICU stay, no (%)	8 (40)
Duration of invasive mechanical ventilation, days	9.1 ± 5.7
Acute Respiratory Distress Syndrome (ARDS)*, no (%)	
Mild ARDS	1 (5)
Moderate ARDS	1 (5)
Severe ARDS	4 (20)
No ARDS	14 (70)
Vasopressors, no (%)	
Noradrenaline	17 (85)
Vasopressin	1 (5)
Noradrenaline + Vasopressin	1 (5)
None	1 (5)
Inotropes**, no (%)	6 (30)
Mechanical circulatory support (ECMO, Impella, IABP), no (%)	1 (5)
Acute Kidney Injury (AKI)***	
AKI grade 1	1 (5)
AKI grade 2	2 (10)
AKI grade 3	5 (25)
Continuous Renal Replacement Therapy (CRRT)	6 (30)
Weight at hospital admission, kg	88.7 ± 21.4
Maximum weight during ICU stay, kg	93.9 ± 23.6
Minimum weight during ICU stay, kg	86.0 ± 22.4
Corticosteroids during ICU stay, no (%)	15 (75)

Data are presented as mean ± SD or median (Q1-Q3)

*ARDS classification according to Berlin criteria;

** dobutamine or milrinone or levosimendan or adrenaline; IABP: intra-aortic balloon pump;

***AKI classification according to KDIGO

Reasons for prematurely interrupting the study protocol are described in the supplement (Appendix S4). Adverse events during the study are presented in Table S2. Demographic variables in both cohorts are listed in Table S3.

### Intervention

Isotopic enrichment in plasma appeared stable over time, and plasma amino acid concentrations increased similarly in both groups after oral protein (Fig. S2 and S3).

The exercise intervention lasted around 20 min. Perceived exertion was high and comparable (OMNI-RES 8 vs. 8.5, *p* = 0.08), whereas concentric and eccentric power were lower in ICU survivors (Fig. S4 and Table S4).

### Muscle thickness, strength and functional outcomes on the study day

**Fig. 2:**
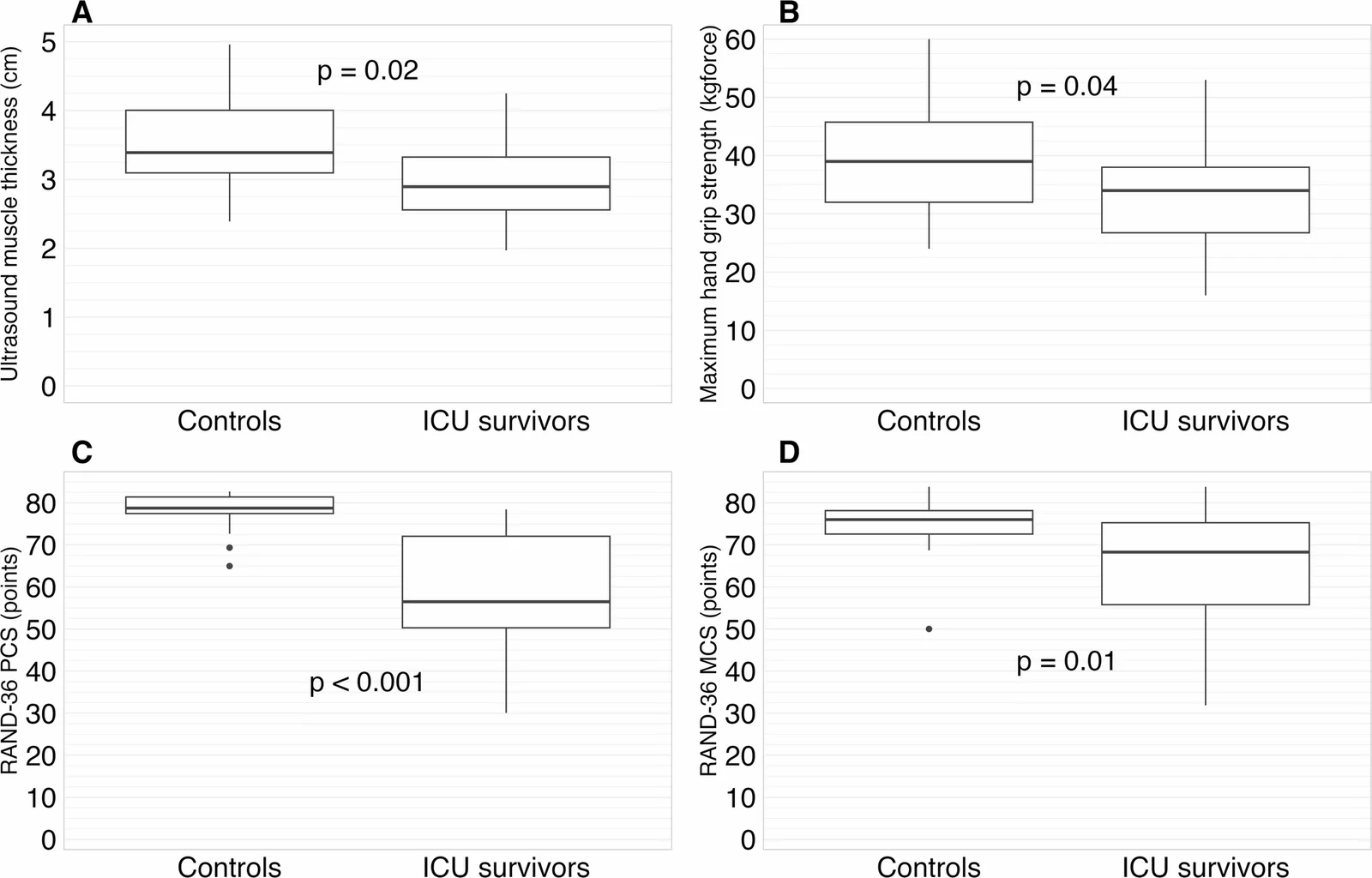
Muscle thickness, strength and health-related quality of life. (**A**) Ultrasound quadriceps muscle thickness (cm), (**B**) Maximum handgrip strength, (**C**) RAND-36 PCS (Physical Component Summary score) and (**D**) RAND-36 MCS (Mental Component Summary score) on the study day. 20 controls/20 ICU survivors

Quadriceps muscle layer thickness (QMLT) and handgrip strength (HGS) were lower in ICU survivors compared to controls (QMLT 2.99 ± 0.65 vs. 3.49 ± 0.66 cm, *p* = 0.02; HGS 32.9 ± 8.9 vs. 39.4 ± 10.4 kg, *p* = 0.04; Fig. 2 and Table S5). ICU survivors had a higher functional comorbidity index and clinical frailty score, and scored lower on both physical (PCS) and mental (MCS) composite scores of RAND-36 (PCS 56.5 [50.3–72.1] vs. 78.8 [77.5–81.4], *p* < 0.001; MCS 68.3 [55.8–75.3] vs. 76.0 [72.6–78.2], *p* = 0.01; Fig. 2 and Table S5).

### Muscle protein synthesis

**Fig. 3:**
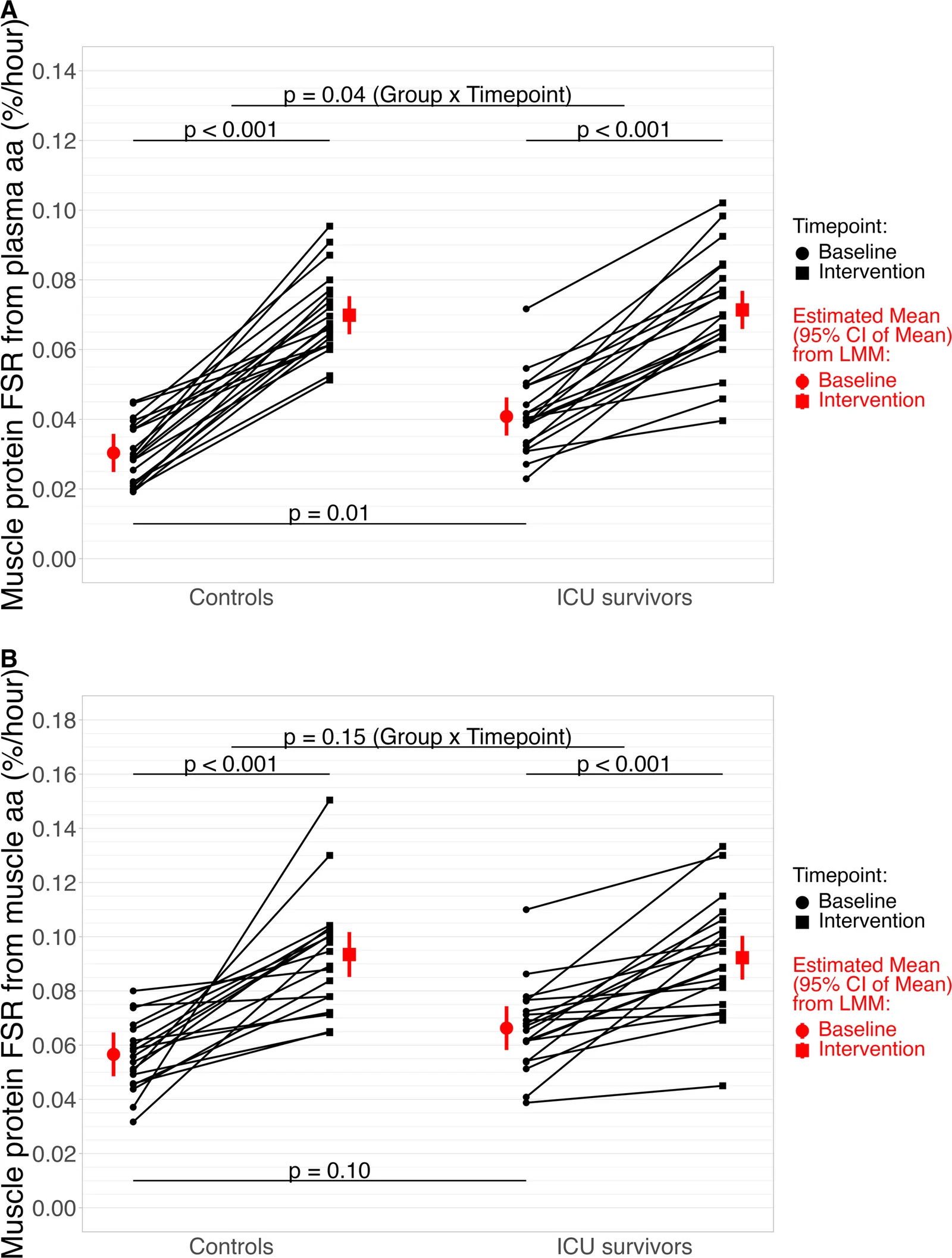
Fractional synthetic rate (FSR) for skeletal muscle proteins from (**A**) plasma or (**B**) muscle precursor amino acids in controls (*n* = 20, *n* = 19 post-intervention in 3B) and ICU survivors (*n* = 20). “Intervention” denotes the measurements from the post-intervention muscle biopsy. aa: precursor amino acids

Muscle protein FSR from plasma precursor amino acids was higher at baseline in ICU survivors (0.0408, 95% CI 0.0353–0.0463%/hour) than in controls (0.0303, 95% CI 0.0249–0.0358%/hour) (*p* = 0.009), but increased less after the intervention (Δ 0.0306, standard error (SE) 0.003%/hour vs. Δ 0.0395, SE 0.003%/hour) (*p* = 0.04). Both groups reached similar muscle protein FSR after the intervention (0.0714, 95% CI 0.0659–0.0768%/hour in ICU survivors vs. 0.0699, 95% CI 0.0644–0.0753%/hour in controls; *p* = 0.696; Fig. 3A and B). Muscle protein FSR from intracellular muscle precursor amino acids did not differ at baseline between ICU survivors (0.0663, 95% CI 0.0582–0.0743%/hour) and controls (0.0566, 95% CI 0.0485–0.0647%/hour) (*p* = 0.10) and increased similarly in both groups post-intervention (Δ 0.0260, SE 0.005%/hour in ICU survivors vs. Δ 0.0369, SE 0.005%/hour in controls) (*p* = 0.15). Raw means ± SD are presented in Table S6.

### Muscle protein synthesis markers

At baseline, anabolic signalling did not differ between groups (Fig. S5), reflected by similar phosphorylated S6/total S6 ratios in controls and in ICU survivors (0.48 vs. 0.10, *p* = 0.53) and similar phosphorylated eEF2/total eEF2 ratios (0.49 vs. 0.44, *p* = 0.55). The intervention increased phosphorylated S6/total S6 to a similar extent in both groups (Δ 4.28 in controls vs. Δ 4.30 in ICU survivors, *p* = 0.98) with no between-group difference in phosphorylated eEF2/total eEF2 ratios (Δ -0.12 in controls vs. Δ -0.23 in ICU survivors, *p* = 0.30).

### Muscle protein breakdown markers

#### Autophagy-lysosomal pathway

**Fig. 4:**
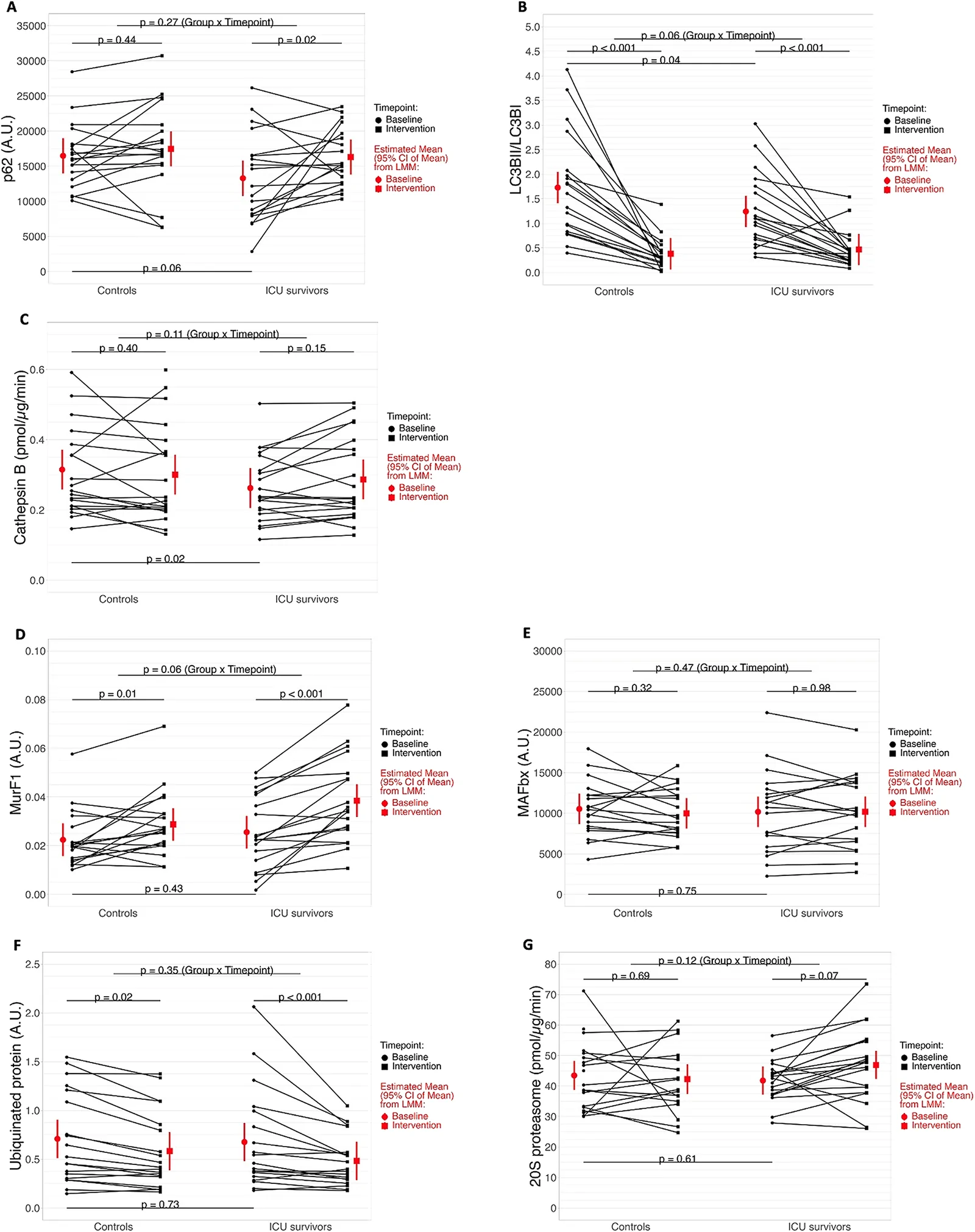
Protein breakdown pathway activity. Panels **A**-**C**: Autophagy-lysosomal pathways. Panels **D**-**G**: Ubiquitin-proteasome pathway. **A**-**B**, **D**-**F**: controls *n* = 19, ICU survivors *n* = 19; C: controls baseline *n* = 19, post-intervention *n* = 20, ICU survivors *n* = 20; G: controls baseline *n* = 19, post-intervention *n* = 18, ICU survivors *n* = 20. “Intervention” denotes the measurements from the post-intervention muscle biopsy. LC3: Microtubule-associated protein 1 light chain 3. MuRF1: Muscle Ring Finger 1, MaFbx: muscle atrophy F-box (also called atrogin-1)

At baseline, ICU survivors had lower autophagy-lysosomal pathway activation, including p62 (13287 vs. 16482 A.U., *p* = 0.06), LC3BII/LC3BI ratio (1.24 vs. 1.73, *p* = 0.04) and cathepsin B activity (0.26 vs. 0.32 pmol/µg/min, *p* = 0.02) (Fig. 4). The intervention reduced LC3BII/LC3BI in both groups with no statistically significant between-group difference (Δ -1.35, SE 0.21 in controls vs. Δ -0.77, SE 0.21 in ICU survivors, *p* = 0.06).

#### Ubiquitin-proteasome pathway

Baseline ubiquitin-proteasome pathway did not differ between groups, including MuRF1, MaFbx, ubiquitinated protein, and 20 S proteasome activity (all *p* ≥ 0.43) (Fig. 4). The intervention increased MuRF1 (Δ 0.006, SE 0.002 in controls vs. Δ 0.013, SE 0.002 A.U. in ICU survivors, *p* = 0.06) and reduced ubiquitinated protein (Δ -0.13, SE 0.05 vs. Δ -0.19, SE 0.05 A.U., *p* = 0.35) similarly in both groups, without affecting MaFbx and 20 S proteasome activity. In presence of the specific proteasome inhibitor MG132 the degradation of the substrate used in our assay is mainly driven by calpain. This non-proteasome activity was lower in ICU survivors than controls at baseline (17.0 vs. 20.3 pmol/µg/min, *p* = 0.03) (Fig. S6). The intervention increased non-proteasome activity only in ICU survivors (Δ 3.3, SE 1.1 vs. Δ 0.2, SE 1.2 in controls, *p* = 0.04).

## Discussion

In this study, we compared the muscle protein synthetic response to a single bout of strenuous resistance exercise and protein ingestion between ICU survivors recovering from multiple organ failure and age- and sex-matched controls. The intervention increased muscle protein synthesis and activated anabolic signalling in both groups. The increase in plasma-precursor FSR from baseline to post-intervention was smaller in ICU survivors than in controls, but this reflected a higher baseline FSR; post-intervention FSR was similar between groups. This suggests that maximal skeletal muscle anabolic capacity is preserved during post-ICU recovery. The post-exercise increase in muscle protein synthesis was similar in magnitude to that observed in a pooled meta-analysis of comparable investigations in healthy volunteers [13].

Results using muscle precursor enrichment were directionally consistent with the plasma precursor findings but not statistically significant. While plasma precursor enrichment provides a more stable estimate due to sampling frequency, muscle precursor enrichment more closely reflects precursor availability at the tissue level. Neither method is clearly superior in terms of reproducibility and yield systematically different results [14]. For this reason, both are presented here without a predefined hierarchy. The concordant pattern across both approaches supports a genuine baseline difference in muscle protein synthesis during post-ICU recovery.

Higher baseline FSR in ICU survivors could reflect increased turnover or a more favourable protein balance during recovery. To explore this, we analysed both protein synthesis markers and proteolysis via the autophagy-lysosomal and ubiquitin-proteasome pathway at baseline and after intervention. At baseline, ICU survivors had lower cathepsin B activity, p62, LC3BII/LC3BI ratio. Markers of the ubiquitin-proteasome pathway did not differ from controls. These findings may indicate reduced autophagy and proteolytic activity, consistent with a more positive protein balance rather than higher turnover. The intervention activated anabolic signalling and reduced initiation of autophagy in both groups, consistent with preserved anabolic responsiveness. In sum, these findings support the post-intervention increase in muscle protein FSR in both groups.

Interestingly, the intervention enhanced components of the ubiquitin-proteasome pathway in both controls and ICU survivors and increased non-proteasome activity, potentially reflecting calpain activity, in ICU survivors. In other studies, resistance exercise initially stimulated an increase in protein breakdown, but to a lesser extent than the increase in protein synthesis [15].

The intervention was designed to elicit a maximal anabolic response: the flywheel ergometer enabled high-intensity eccentric loading, demonstrated to promote both acute increases in muscle protein synthesis and long-term hypertrophy [16]. Subjects were actively coached to fatigue themselves during the training sets by supervising staff, and perceived exertion was high in both groups. The whey protein dose was above the established maximal effective amount for stimulating muscle protein synthesis in healthy individuals [17]. We therefore cannot exclude that ICU survivors have a higher anabolic threshold, and may respond less to submaximal stimuli. This could partially explain the mixed signals observed in trials investigating post-ICU rehabilitation programmes [9].

From a clinical standpoint, these results support the rationale for structured resistance exercise combined with adequate protein provision in post-ICU rehabilitation. How protein should optimally be delivered in this context remains to be determined. In healthy adults, 20 g of whey protein every 3 h after resistance exercise sustained myofibrillar protein synthesis more effectively than larger, less frequent or smaller, more frequent feedings [18]. Whether a similar nutritional strategy could enhance the anabolic response in ICU survivors is an interesting question for future research.

### Context to previous literature in critically ill patients

The only study investigating an intervention’s direct effects on muscle anabolism is by Chapple et al. [6]. Using similar methods, they found a blunted anabolic response to duodenal protein in mechanically ventilated ICU patients compared to controls. In contrast, our study showed a far more pronounced anabolic response during post-ICU recovery, when protein intake was combined with strenuous exercise. The impact of exercise on muscle protein balance during intensive care remains an open question. However, biomechanical and volitional constraints may limit whether feasible ICU-based interventions can generate sufficient loading conditions to further stimulate protein synthesis or muscle growth [19].

Translational studies of post-ICU muscle function have identified potential mechanisms behind persistent muscle weakness that may not be amenable to anabolic stimulation from exercise. These include epigenetic modifications affecting muscle regeneration, altered gene transcription regulating fibrosis and adipose tissue and reduced satellite cell populations [8, 20, 21]. Chronic remodelling with extracellular matrix deposition and myosteatosis, together with impaired regeneration of myocytes from stem cells, could partly explain the plateau in physical recovery 6 to 12 months after critical illness. The intervention in our study was scheduled 3 to 6 months after ICU discharge to coincide with the period where physical function is known to improve [3]. As immunohistochemistry and transcriptomics analyses are pending, we cannot determine if similar changes in muscle architecture are present in this cohort. Future studies should address whether there is an optimal window and optimal training form to rebuild functional muscle mass before chronic degenerative changes occur.

Regardless, our results provide optimism regarding the potential for rehabilitation of muscle function after critical illness. Current mechanistic data from the post-ICU period paint a bleak picture of recovery potential, frequently highlighting future precision medicine approaches like stem cell injections or microRNA therapeutics [22, 23]. In contrast, this study suggests an intact response to conventional anabolic interventions.

### Strengths and limitations

Our study has several strengths. To our knowledge, this is the first study to investigate the direct anabolic effects of resistance exercise and protein intake in former ICU patients. We enrolled a cohort of ICU survivors with high illness severity during hospitalisation, including multiple risk factors for ICU-acquired weakness [24]. The distribution of age, sex and admission diagnoses was representative of a general adult ICU population, strengthening external validity. By prospective 1:1 enrolment of age- and sex-matched controls, we also minimised confounding effects of baseline demographic differences between groups [25]. The tracer incorporation technique used in our study is the most direct method available to assess the acute anabolic response to exercise. Isotopic steady state was monitored by blood sampling at regular intervals and maintained by an oral tracer during protein ingestion.

Several limitations should be considered. Participation in the study was time-consuming and included potentially painful procedures. Despite systematic screening, this introduced the risk of selection bias, as participants may have had unmeasured baseline physical and/or mental characteristics not representative of the target population. However, RAND-36 physical and mental component summary scores were nearly identical to those observed in a recent nationwide cohort study of ICU patients with sepsis [26]. Differences in muscle mass and strength were also similar to those described in a one-year follow-up of critically ill patients using age-matched controls [27].

We did not document a detailed history of physical activity prior to participation. Although participants were instructed to avoid exercising one day prior to the protocol, residual cellular effects of exercise in individuals cannot be excluded.

Muscle protein breakdown was not measured due to the highly invasive and complex methodology required. Therefore we cannot determine the impact of the intervention on net protein balance. Instead, pathway markers and enzymatic activities at baseline and post-intervention were analysed as surrogate measures of proteolysis.

Due to standardised sample preparation for all analyses, we measured mixed muscle rather than myofibrillar protein synthesis. As the latter is more specific to the contractile adaptation targeted by exercise, a differential response between the groups cannot be excluded.

The precursor pool used to calculate muscle protein FSR was not specified a priori. Although this introduces the potential for selective reporting, estimates based on both precursor pools were directionally consistent and are presented as complementary analyses.

As we only measured acute effects, we cannot exclude an attenuation of the prolonged anabolic response over 24 to 48 h in the post-ICU phase [28].

## Conclusions

Compared with matched controls, ICU survivors recovering from critical illness have a smaller incremental response in muscle protein synthesis to combined strenuous resistance exercise and protein intake, in the context of a higher baseline synthetic rate. The post-intervention synthetic rate was similar between groups, indicating a preserved maximal anabolic capacity. These findings provide a biological rationale for exercise-nutrition strategies to promote muscle recovery after ICU discharge. Further research is needed to clarify the long-term effects on recovery of muscle mass and function, and to define the optimal dose and timing of interventions.

## Supplementary Information

Below is the link to the electronic supplementary material.

Supplementary Material 1

Supplementary Material 2

## Data Availability

Due to confidentiality, patient-level data will not be made available. De-identified and anonymized data for individual analyses may be shared on reasonable request, after subject to applicable ethical and data-sharing approvals.

## Abbreviations

ICU: Intensive care unit
ICUAW: Intensive care unit-acquired weakness
FSR: Fractional synthetic rate
SAPS 3: Simplified acute physiology score 3
SOFA: Sequential organ failure assessment
FCI: Functional comorbidity index
OMNI-RES: OMNI perceived exertion scale for resistance exercise
pS6/tS6: Ratio of phosphorylated to total protein S6
peEF2/teEF2: Ratio of phosphorylated to total eukaryotic elongation factor 2
LC3: Microtubule-associated protein 1 light chain 3
MuRF1: Muscle Ring Finger 1
MaFbx: Muscle atrophy F-box (also called atrogin-1)
