# Supplementary material for: Preserved anabolic skeletal muscle capacity in response to exercise and protein intake after critical illness
Source: Crit Care. 2026 Oct 24;30:549. doi: 10.1186/s13054-026-06418-7 (PMC13700533; doi:10.1186/s13054-026-06418-7)
Supplement: Supplementary file 1 [file 13054_2026_6418_MOESM1_ESM.pdf]

Supplementary Data

Preserved anabolic skeletal muscle capacity in response to exercise and protein intake after critical illness

Arabella Fischer-Hammerschmied[1,2], Nicolas Tardif[1], Mirko Mandić[3,4], Thomas Gustafsson[3,4], William Apro[5], Johan Helleberg[1,6], Mariam Amouzandeh[1,6], Jonathan Grip[1,6], Dag Seldén[1,6], Olav Rooyackers[1,6#], Martin Sundström Rehal[1,6#]

[1] Division of Anaesthesia and Intensive Care, Department of Clinical Science, Intervention and Technology (CLINTEC), Karolinska Institutet, Stockholm, Sweden

[2] Division of Cardiothoracic and Vascular Anaesthesia and Intensive Care Medicine, Department of Anaesthesia, Intensive Care and Pain Medicine, Medical University of Vienna, Austria

[3] Department of Laboratory Medicine, Division of Clinical Physiology, Karolinska Institutet, Stockholm, Sweden

[4] Department of Clinical Physiology, Karolinska University Hospital, Stockholm, Sweden

[5] Department of Physiology, Nutrition and Biomechanics, Swedish School of Sport and Health Sciences, GIH, Stockholm, Sweden.

[6] Department of Perioperative Medicine and Intensive Care (PMI), Karolinska University Hospital Huddinge, Stockholm, Sweden

Corresponding author: Martin Sundström Rehal

Mailing address: K32 Funktion PMI, Hälsovägen 13, Karolinska University Hospital Huddinge, 14186, Stockholm Sweden

E-mail: martin.sundstrom.rehal@ki.se

Phone number +46-8-12381507

[#] Contributed equally.

Table of contents

Table S1. Detailed exclusion criteria for ICU survivors and controls
Appendix S1. Screening and inclusion process
Figure S1. Inclusion flowchart for ICU survivors
Appendix S2. Experimental protocol
Appendix S3. Analytical techniques
Appendix S4. Reasons for prematurely interrupting the study protocol
Table S2. Adverse events of all participants who started the study and number of participants on anticoagulant medications during the study day.
Table S3. Comparison of demographic variables between ICU survivors and controls
Figure S2. Plasma tracer enrichment
Figure S3. Plasma amino acid concentrations before and after the oral protein drink
Figure S4. Peak power during exercise.
Figure S5. Protein synthesis markers
Figure S6. Non-proteasome activity
Table S4. Resistance exercise
Table S5. Muscle thickness, strength and functional outcomes on the study day
Table S6. Raw means and standard deviations for muscle protein FSR
References

Table S1. Detailed exclusion criteria for ICU survivors and controls

**All participants**
Inability to provide informed consent
Warfarin or ADP receptor antagonists
Clinically significant congenital or acquired coagulopathy
Lower-leg amputation
Critical ischaemia of the lower extremity
Fracture or osteoarthritis with marked limitation of motion in the hip or knee joint
Metastatic cancer or active haematological malignancy
Congenital disorder of amino acid metabolism
Chronic muscular or neurological disease with previously documented weakness in the lower extremities
Pregnancy
Raynaud syndrome
**ICU survivors only**
Less than two organ failures if not intubated for respiratory failure
Intubated only for airway protection with no other organ failure(s) during ICU stay
Planned postoperative care after cardiac surgery

Appendix S1. Screening and inclusion process

The first cohort included former adult patients admitted to one of the hospital's intensive care units. Between March 2022 and November 2023, all admissions registered in the patient data management system with an ICU length of stay of at least 72 hours from the preceding three-month period were regularly screened. Former patients could be included within three to six months from ICU discharge. If no exclusion criteria were identified from screening of the electronic medical records, ICU survivors who had returned home were contacted by telephone. In the event of limited resources for screening, priority was given according to length of ICU stay in descending order. All phone calls were conducted by the primary investigator (MSR) and included standardised information about the study aims, conduct and potential risks as described in the participant information sheet approved by the Swedish Ethical Review Authority. In cases where study participation was perceived not to be in the best interest of former patients (for example due to intense medical follow-ups, severe psychological distress or substance abuse), the PI could refrain from contacting potential participants based on clinical and ethical judgements. If a former ICU patient had ongoing outpatient care, the responsible physician was identified and contacted to ensure that no disease-specific risks for study participation were present.

The second cohort of non-critically ill controls was recruited through public advertisements posted in the hospital area. Controls were eligible if they had previously not been hospitalised in an ICU. Exclusion criteria in Table S1 also applied to controls. Study participation for the control group was conducted in parallel with the post-ICU group, depending on matching criteria and availability.

Study days were scheduled according to availability and convenience of the participants, within the given time frame. As the study required spending a full working day at the study site, all participants were compensated with SEK 2,000 (approximately EUR 180) in accordance with the approved ethical application. A late lunch was provided after completion of the protocol, and transportation to and from the study site was arranged by the investigators.

Figure S1. Inclusion flowchart for ICU survivors

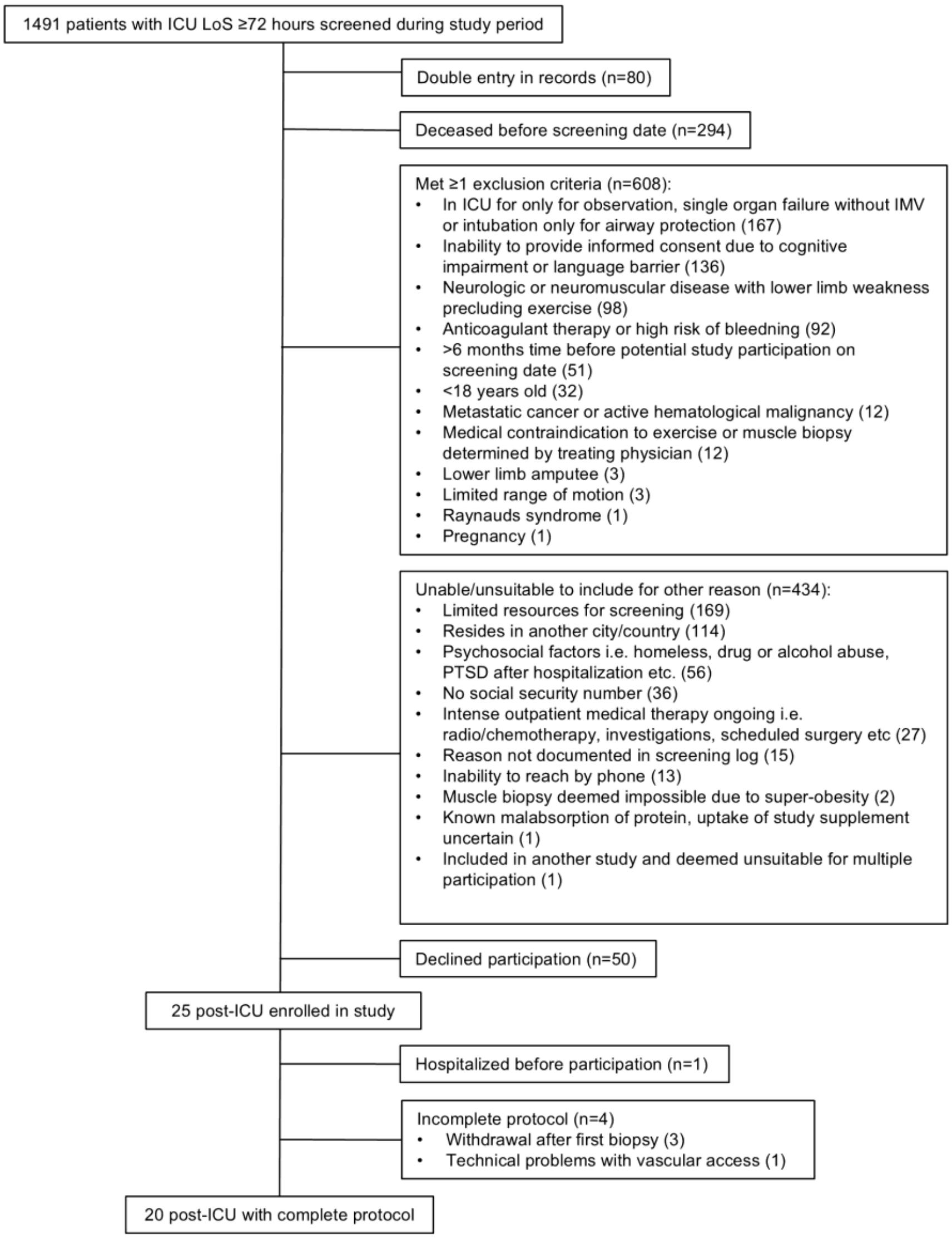

Appendix S2. Experimental protocol

The protocol was identical in both cohorts. Participants were instructed to fast from 22:00 before study participation, allowing water or other non-caloric, protein-free beverages, and to avoid exercise on the preceding day. Dietary intake prior to fasting was not standardised. No medications were discontinued on the study day.

After arrival to the study site (Karolinska University Hospital Huddinge) in the morning, the participant's weight was measured on a standing scale, and a peripheral venous catheter was sited primarily in the non-dominant arm. After assessing collateral blood flow an arterial line was placed in the radial artery under local anaesthesia using ultrasound guidance. After baseline blood samples, a primed intravenous infusion of $^{2}H_{5}$-phenylalanine (bolus 2.94 µmol/kg, infusion rate 2.94 µmol/kg/h) was started to achieve steady-state distribution of tracer between plasma and the intracellular precursor pool, and continued until the end of the study. During the study protocol arterial blood samples were drawn every 15 minutes to determine tracer enrichment and amino acid concentrations over time.

After 150 minutes of tracer infusion, a baseline muscle biopsy was extracted from the vastus lateralis muscle of the non-dominant leg using a Bergström needle under fully sterile conditions. Local anaesthesia was applied to the skin and above the muscle fascia. Arterial blood samples were drawn simultaneously. Skin closure was performed with sterile tape covered with an adhesive dressing, and manual compression was applied before wrapping the thigh with an elastic bandage.

Immediately after the first muscle biopsy, resistance exercise was performed with the dominant leg in a flywheel leg extension machine (YoYo Technology™) under study team guidance. The flywheel device stores energy produced in the concentric movement to be counteracted in the eccentric phase, offering brief episodes of eccentric overload that have

been shown to promote muscle hypertrophy [1]. After an introduction and three warm-up sets, participants completed four sets of 10 leg extensions with a three-minute rest between sets. Subjective effort immediately after the last set was rated with the OMNI Perceived Exertion Scale for Resistance Exercise (OMNI-RES) scale graded 0-10.

Upon completion of the exercise, participants received 24 g of hydrolysed protein (30 g of Whey Protein Hydrolysate, Body Science™). The rationale, dosing and timing of protein ingestion were based on multiple prior observations that concurrent protein intake enhances the anabolic response to resistance exercise [2-4]. The powder was weighed on a precision scale and blended in approximately 300-400 ml of water. To avoid dilution of the tracer plasma pool, 40 mg of $^{2}H_{5}$-phenylalanine was added to the protein beverage prior to ingestion, with a resulting enrichment (molar percent excess) of 6.34%. The mixed beverage was provided in portions to drink over 5 minutes timed with a stopwatch. Residue from the drink in the shaker was mixed with additional water at the end to ensure that a standardised amount was consumed. After a 150-minute resting period starting at the beginning of oral protein intake, a second muscle biopsy was extracted from the vastus lateralis muscle of the dominant leg. The tracer infusion was then stopped and all catheters removed.

Any adverse events related to study interventions were documented in the case report form. All participants were monitored for an hour before returning home, and received written information detailing wound care after biopsies, monitoring for potential complications and contact information to the investigators in case a follow-up was requested.

Body composition and handgrip strength measurements

Weight was measured on a standing scale. Quadriceps muscle thickness was measured on the dominant leg at the midpoint of the thigh according to the USVALID protocol [5]. The participant was placed in a semi-recumbent position with the head of the bed at 30 degrees, a slight outward rotation of the leg and a foam roll supporting the knee. The middle of the

femur was identified at half the distance between the upper border of the greater trochanter and the lateral anterior knee joint space. The middle of the quadriceps muscle was identified at the intersection between the mid-femur point and a line between the anterior superior iliac spine and the patella. After marking this point, a single ultrasound measurement with a linear 4-13 MHz probe (Clarius Mobile Health, Vancouver, Canada) was performed in the axial plane using at least 1 cm of ultrasound gel to ensure minimal compression. After image acquisition, a line was drawn from the middle of the image at the level of the skin to the border of the femur with the shortest possible distance. Muscle thickness was measured along this line from just below the muscle fascia to the bone surface. Handgrip strength was measured with the participant seated, a ~90 degree bend at the elbow and the forearm supported on a table. After instructions, two measurements were recorded for each hand with the highest value entered in the case report form.

Training equipment and exercise programme

While seated in a slightly reclined position with back support, the participant pushed against a crossbar located at the distal end of a pivoting arm, whose axis of rotation was aligned with the knee joint. Starting from a knee angle of approximately 90°, the flywheel began to rotate by pulling a strap attached to both the flywheel shaft and the distal end of the lever arm, looping around a curved cam. As the participant extended the knee, the strap unwound from the flywheel shaft, transferring force and energy to the flywheel. Upon reaching about 170° of knee extension, the pushing concentric phase ended, and the strap rewound due to the kinetic energy stored in the flywheel, pulling the lever arm back. As the flywheel pulled the strap, the participant resisted the force, performing an eccentric muscle action. The selected exercise modality enabled participants to exert maximum intensity on each repetition, making it highly effective. Previous studies from our consortium have shown that flywheel knee-extension exercise elicits marked neuromuscular fatigue at a dose similar to the current protocol, and when performed repeatedly, produces gains in muscle mass and strength comparable to conventional heavy resistance exercise [1, 6-7]. Therefore,

participants in this study were subjected to an exercise known to be potent enough to stimulate long-term muscle growth and, consequently, trigger acute increases in protein synthesis.

Appendix S3. Analytical techniques

Blood samples were drawn in EDTA tubes, stored on ice, centrifuged within 60 minutes and stored at -80°C until analyses. Muscle biopsies were immediately frozen in liquid nitrogen and stored at -80°C until analyses.

The muscle samples were freeze-dried and pulverised. Fat, connective tissue, and blood were carefully removed from the muscle fibres. Freeze-dried samples were weighed and divided into aliquots for subsequent protein synthesis measurement, proteolytic activity assays, and western blot analysis. Tracer enrichment in plasma and muscle specimens was determined by gas chromatography-mass spectrometry, and FSR calculated from the ratio of enrichment in mixed muscle protein and the plasma and free muscle precursor pools according to the equation:

$$FSR\ (\% * h^{-1}) = \frac{E_{p,t} - E_{p,0}}{E_{precursor} * t} * 100$$

Where $E_{p,t}$ is the protein-bound enrichment in the biopsy at time t, $E_{p,0}$ is the protein-bound enrichment at baseline, $E_{precursor}$ is the mean value of plasma enrichment over the incorporation period or the muscle tissue free $^{2}H_{5}$-phenylalanine enrichment (depending on precursor pool used in calculation), and t is the incorporation time. For the baseline FSR calculation $E_{p,0}$ was set to zero as the natural abundance of $^{2}H_{5}$-phenylalanine is negligible. For post-intervention FSR, it is derived from protein-bound enrichment in the first biopsy.

A comprehensive summary of all procedures and calculations is described in a previous publication from our group [8].

Plasma amino acid concentrations were analysed in the baseline samples and every 15 minutes after the intervention using a standard HPLC technique. Due to limited resources, plasma amino acids were analysed in 14 post-ICU participants and 14 controls.

Fluorescence analyses of cathepsin B and 20S proteasome activity

For activity analyses, pre- and post-exercise samples from ICU survivors and their corresponding matched controls were analysed on the same plate. Freeze-dried muscle was homogenised in Mammalian Protein Extraction Reagent (M-PER, ThermoFisher Scientific) without the addition of antiprotease and centrifuged at 1000 × g for 10 minutes at 4°C to clean up homogenates. Muscle homogenate was aliquoted for the assessment of cathepsin B and 20S proteasome activity and stored at -80°C until analysis. One aliquot was used to measure protein concentration with the micro BCA kit (23235, ThermoFisher Scientific) following the provider's protocol. Chymotrypsin-like activity of the proteasome fraction was measured using the fluorogenic substrate SUC-LLVY-AMC [succinyl-Leu-Val-Tyr-(7-amido-4-methylcoumarin)-AMC]. 10μl of muscle homogenate (~10 μg protein) was incubated in 100 μL buffer (50 mM Tris-HCl, pH 7.5, 1 mM ATP, 5 mM MgCl2, 1 mM DTT, and 150 μM LLVY) in microplates. As the substrate SUC-LLVY-AMC can also be degraded by other proteases, mainly calpain in neutral buffer, 20S proteasome-specific activity was measured in the presence and absence of MG132 (10 μM), a specific proteasome inhibitor. Cathepsin B activity was measured using Z-Arg-Arg-AMC (C5429, Merck). 20 μL of muscle homogenate (~20 μg protein) was incubated in 100 μL buffer (0.1 M sodium phosphate, pH 6.0, 1 mM EDTA, 2 mM cysteine, and 250 μM Arg-Arg-AMC). Standard curves were prepared using AMC (A9891, Merck). Fluorescence was measured continuously over 1 h at 37°C in a CLARIOstar Plate Reader (BMG Laboratory Technologies) at λex = 380 nm and λem = 460 nm. Proteolytic activity was calculated from the increment of the curves from samples and standards and is expressed as pmol of AMC released per μg protein per minute.

Western blots of p62, LC3 I, LC3 II, MuRF1, MAFbx, eEF2, S6 proteins

Muscle samples were homogenised in ice-cold lysis buffer (0.2 ul/ug) using a Bullet Blender (Next Advance, USA) and 0.5 mm Zirconium Oxide beads. The lysis buffer had a final concentration of 40 mM HEPES (pH 7.5), 1 mM EDTA, 120 mM NaCl, 10 mM β-glycerophosphate, 10 mM Na4P2O7, 50 mM NaF, 0.25% Triton X 100, 1% phosphatase inhibitor cocktail (Sigma P0044), and 1% (vol/vol) Halt Protease Inhibitor Cocktail (Thermo Scientific, Cat# 78430). Following 8 × 1 min in the Bullet Blender, samples were shaken (2200 rpm) for 1 h on an orbital shaker. The crude lysate was then centrifuged at 10,000 g for 10 min to generate a supernatant. Protein concentration was determined in the supernatant using Pierce 660 nm protein assay (Thermo Scientific, Cat# 22662). Lysis buffer and 4× Laemmli sample buffer (Bio-Rad Laboratories, Richmond, USA, Cat# 1610747) were combined to adjust the final protein concentration to 0.635 μg · μL^{-1}. Samples were then heated for 30 min at 37°C and subsequently stored at −80°C until analysis.

Samples were loaded (15 ul) in sequential order according to the laboratory ID on 26-well Stain-Free Criterion TGX gradient gels (4%–15% acrylamide; Bio-Rad Laboratories, Cat#5678085). Electrophoresis was performed on ice for ~26 min at 300 V after which the gels were activated with UV light for 45 seconds in the ChemiDoc™MP imaging system (Bio-Rad Laboratories). Proteins were then transferred to low-fluorescence 0.2 uM PVDF membranes (Amersham™ Hybond™ P, Cytiva, Cat#10600022) using the Trans-Blot Turbo System (Bio-Rad Laboratories). Successful transfer was then confirmed by stain-free imaging of each membrane.

These were then blocked in EveryBlot blocking buffer (Bio-Rad Laboratories, Cat# 12010020) for 15 minutes at room temperature. Following blocking, membranes were incubated with primary antibodies diluted in blocking buffer overnight at 4 °C. The next day, membranes were washed in TBST (Tris-buffered saline with Tween-20; 20 mM Tris base, 137 mM NaCl, pH 7.6, 0.1% Tween-20 v/v) for 3 x 5 minutes. Next, the membranes were

incubated with secondary antibodies diluted in blocking buffer for 1 hour at room temperature, washed again as described above, and finally incubated in Super Signal™ Femto Chemiluminescent Substrate (Thermo Scientific, Cat# 34095). Bands and whole lanes were visualised using the ChemiDoc™MP imager (Bio-Rad Laboratories). Data acquisition was performed using the Image Lab Software (v 6.1 Bio-Rad Laboratories). Total protein expression, as well as protein phosphorylation, were related to global protein levels obtained by Stain-Free imaging. When applicable, phosphorylated protein levels were subsequently related to corresponding total protein levels.

Primary antibodies against Ubiquitin (#3936, 1:500), LC3b (#43566,1:500), eEF2 (#2332, 1:500), phospho-eEF2 (#2331, 1:1000), S6 (#2217, 1:500) and phospho-S6 (#5364, 1:500) were purchased from Cell Signaling Technology. The primary antibodies against MuRF1 (#sc-398608, 1:500) and MAFbx (ab168372, 1:1000) were purchased from Santa Cruz Biotechnology and Abcam, respectively. The primary antibody against p62 (#610833, 1:500) was purchased from BD Biosciences. Secondary anti-mouse (#7076, 1:10,000) and secondary anti-rabbit (#7074, 1:10,000) were purchased from Cell Signalling Technology.

Appendix S4. Reasons for prematurely interrupting the study protocol

The first participant belonging to the control group was excluded due to subsequent change of the tracer protocol, adding an oral tracer to avoid dilution of the plasma pool. The study was prematurely interrupted in 6 participants, because of subjective pain due to the arterial catheter with no adverse events (1 control), intramuscular haematoma two hours after the first muscle biopsy (1 control), temporary dysfunction of a port-a-cath catheter (1 ICU survivor), pain during the first muscle biopsy (3 ICU survivors).

Table S2. Adverse events of all participants who started the study and number of participants on anticoagulant medications during the study day.

no (%)	Controls (n=23)	ICU survivors (n=24)	All (n=47)
Bruising or haematoma due to arterial line during or after study participation	6	1	7 (15)
Transient digital hypoperfusion due to arterial line after study participation	1	0	1 (2)
Dizziness during study intervention	3	1	4 (9)
Underwent at least first muscle biopsy	22	23	45 (96)
Intramuscular haematoma	1	0	1 (2)
Participant on DOAC	0	3	3 (6)
Participant on low-dose aspirin	1	1	2 (4)

DOAC: direct oral anticoagulant

Table S3. Comparison of demographic variables between ICU survivors and controls

	Controls (n=20)	ICU survivors (n=20)	p
Female, n (%)	6 (30)	6 (30)	1
Age, years [min-max]	58.9 ± 12.5 [28.1-77.3]	59.6 ± 12.4 [28.5-78]	0.88
Height, cm	176 ± 8	176 ± 9	0.89
Weight, kg	80.0 (70.8-83.3)	81.5 (68.8-94.8)	0.64
BMI, $kg.m^{-2}$	23.8 (22.8-26.8)	26.9 (22.9-28.7)	0.42

Data are presented as mean ± SD or median (Q1-Q3).

Figure S2. Plasma tracer enrichment, expressed as molar percent excess (% labeled $^{2}H_{5}$-phenylalanine) along the study day in all 40 participants (20 controls and 20 ICU survivors), mean ± SD. A primed intravenous infusion of $^{2}H_{5}$-phenylalanine was started at time = -150 minutes prior to first muscle biopsy, with blood samples for determination of plasma enrichment drawn at 15 minute intervals. A drink composed of 24 g of hydrolysed protein and 40 mg of $^{2}H_{5}$-phenylalanine was ingested at time "0" minutes.

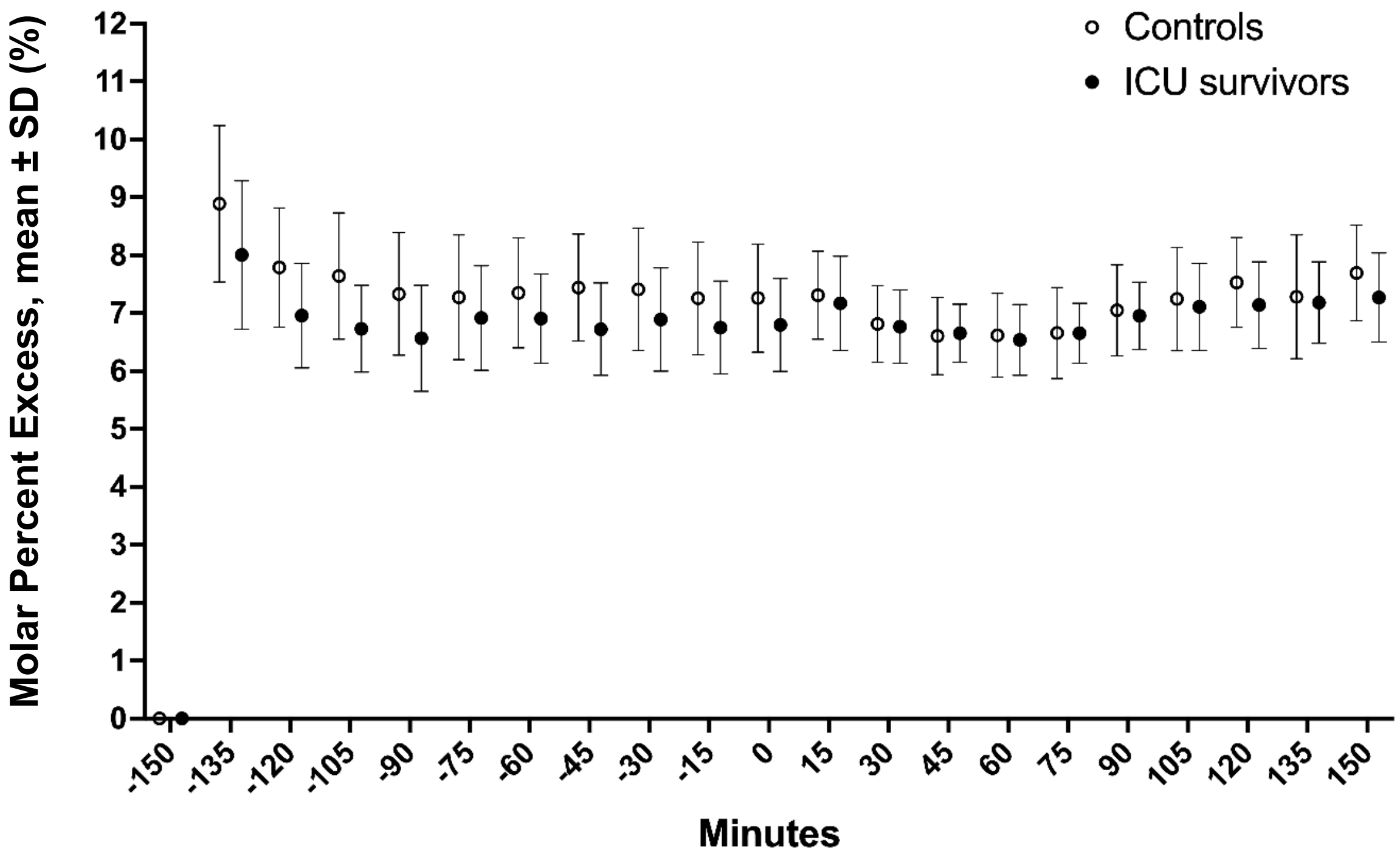

Figure S3. (A) Branched-chain amino acid concentration and (B) Total amino acid concentration (micromol/L) in plasma along the study day before and after the oral protein drink in n=14 controls and n=14 ICU survivors, mean ± SD. Due to limited analysis resources, plasma amino acids were analysed in 14 ICU survivors and 14 controls.

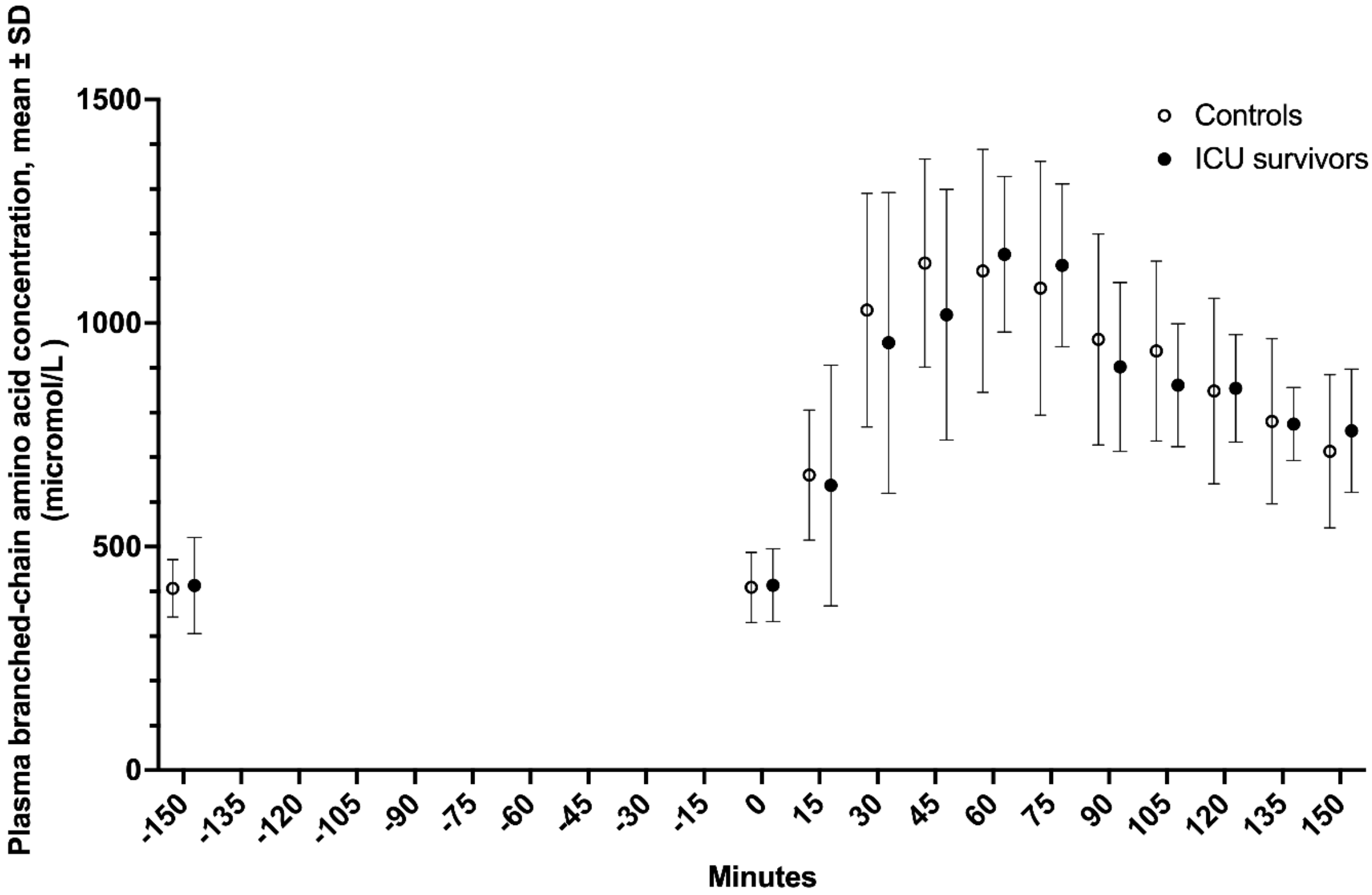

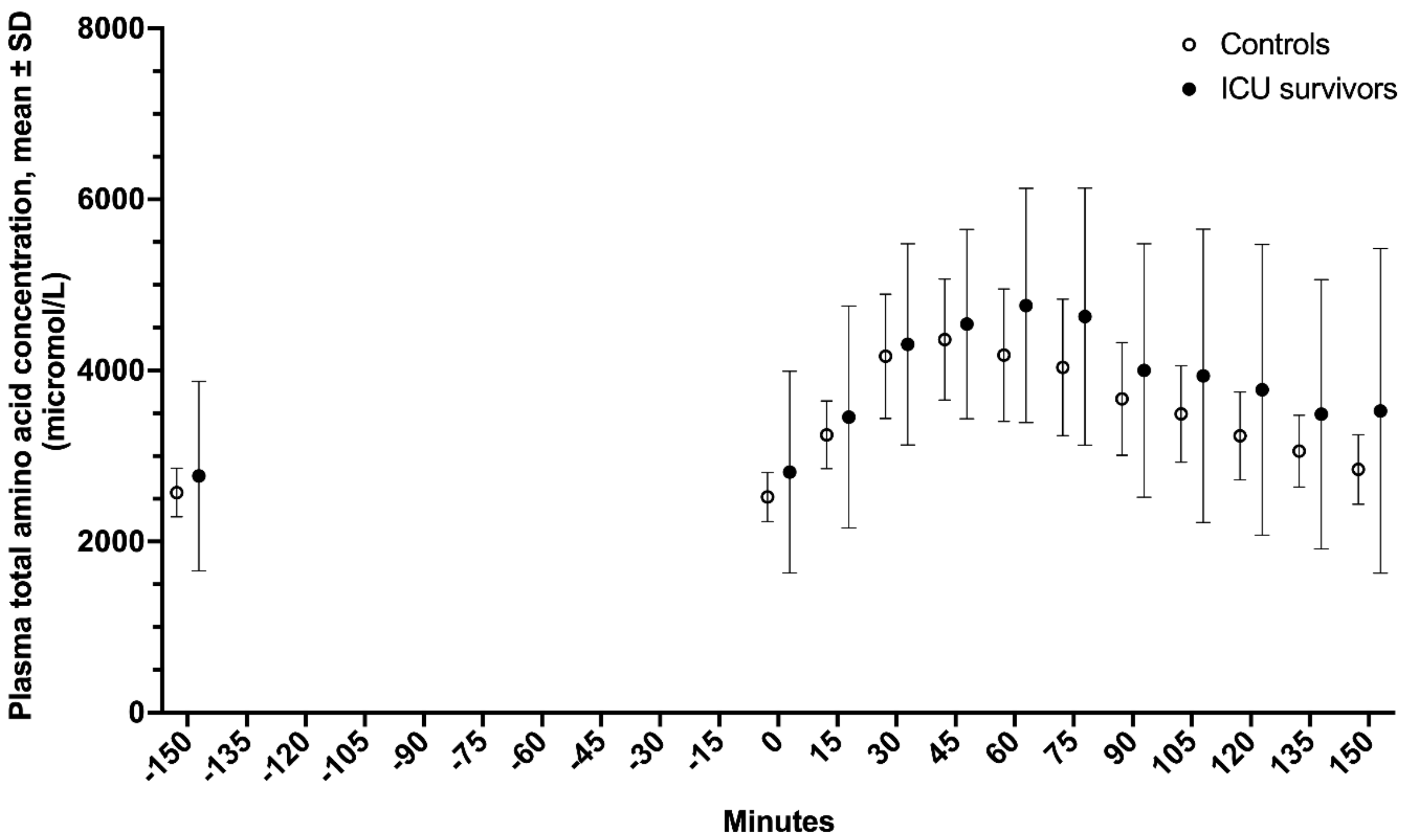

Fixed effects (type III)	P value
Group	0.6526
Time	<0.0001
Group x Time	0.4740

Fixed effects (type III)	P value
Group	0.4302
Time	<0.0001
Group x Time	0.5424

Figure S4. Peak concentric (A) and eccentric (B) power during resistance exercise

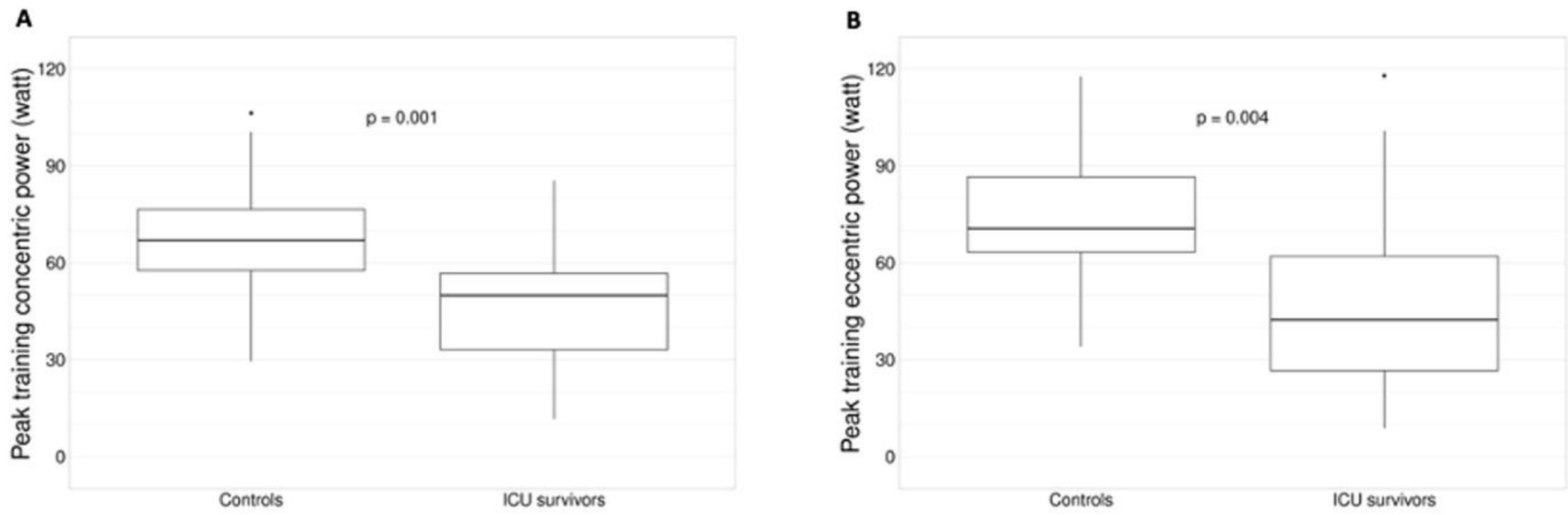

Power data were missing in 2 ICU survivors due to a technical issue with the Flywheel software.

Figure S5. Protein synthesis markers

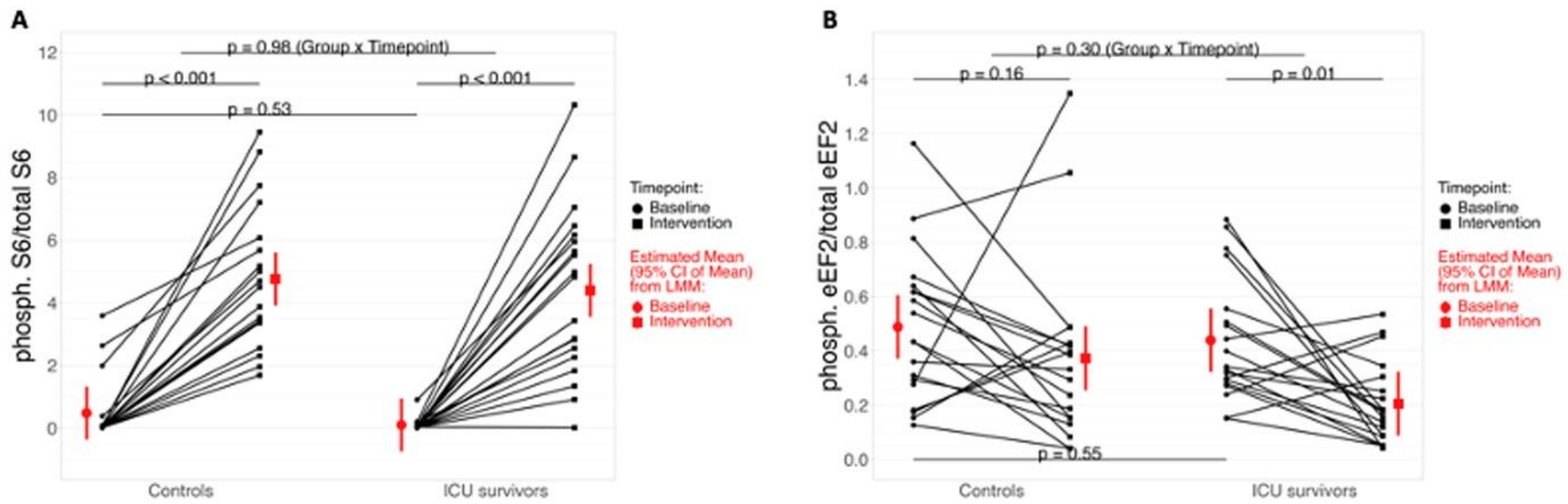

Controls n=19; ICU survivors n=19.

pS6/tS6: ratio of phosphorylated to total protein S6, peEF2/teEF2: ratio of phosphorylated to total eukaryotic elongation factor.

Figure S6. Non-proteasome activity

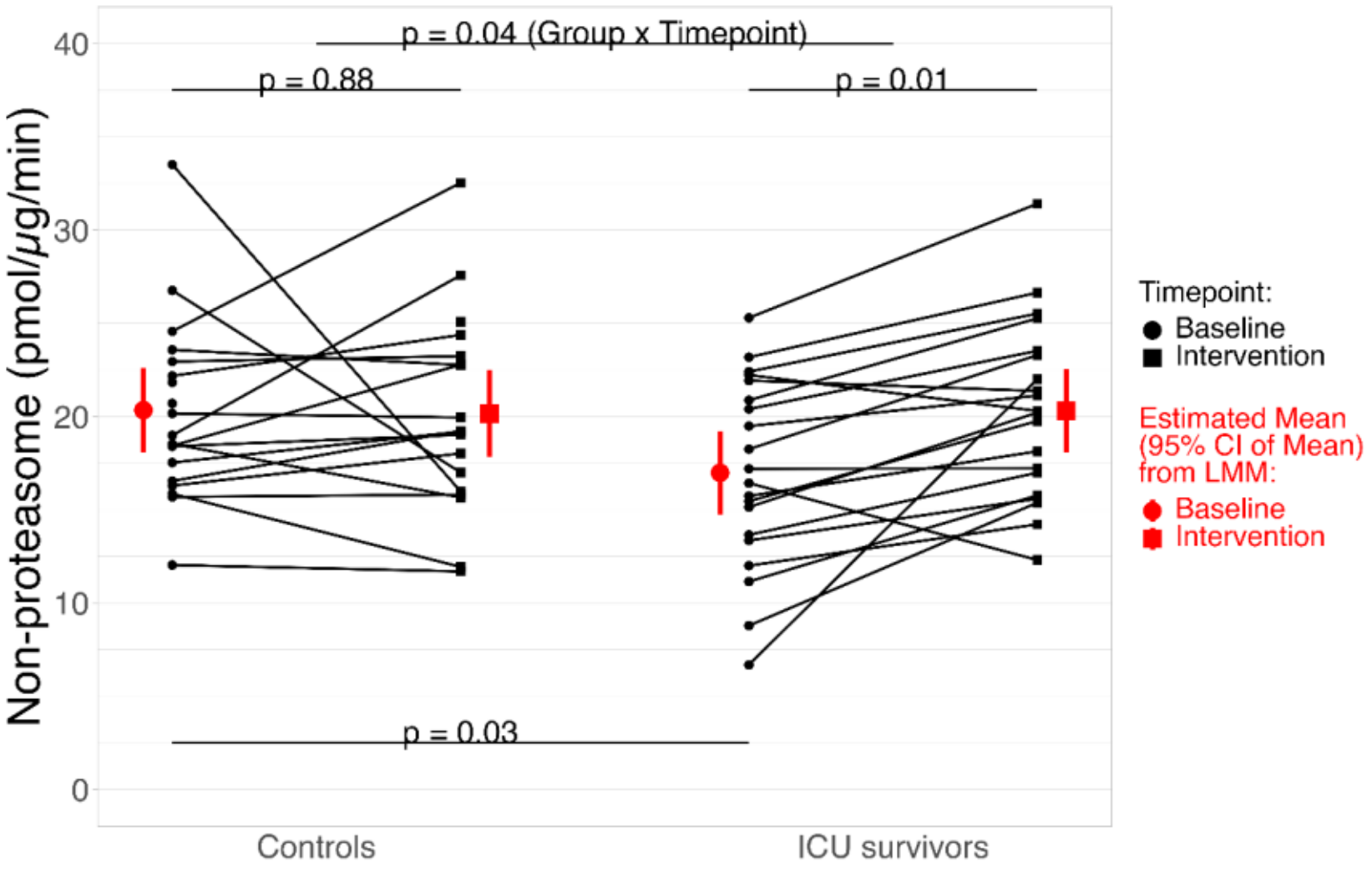

Controls n=19 baseline, n=18 post-intervention; ICU survivors n=20.

Table S4. Resistance exercise

	Controls (n=20)	ICU survivors (n=20)	p
Exercise duration including 2 last warm-up sets and 4 training sets, minutes	20.3 ± 3.1	19.8 ± 2.3	0.56
Inertia of flywheel during warm-up, no. (%)			0.11
0.5	8 (40)	14 (70)	
1	11 (55)	6 (30)	
1.5	1 (5)	0 (0)	
Inertia of flywheel during training, no. (%)			0.52
0.5	10 (50)	13 (65)	
1	9 (45)	7 (35)	
1.5	1 (5)	0 (0)	
**Peak concentric power during warm-up, watt**	62.6 ± 16.6	44.7 ± 20.6	**0.005**
**Peak eccentric power during warm-up, watt**	64.0 ± 30.0	42.6 ± 25.2	**0.02**

**Peak concentric power during training, watt**	68.9 ± 18.7	47.2 ± 19.9	**0.001**
**Peak eccentric power during training, watt**	74.9 ± 22.1	48.8 ± 29.4	**0.004**
Subjective effort of last training set on OMNI-RES scale, points scale: 0-10	8.5 (8-9)	8 (7-8.3)	0.08

Table S5. Muscle thickness, strength and functional outcomes on the study day

	Controls (n=20)	ICU survivors (n=20)	p
**Ultrasound thigh muscle thickness, cm**	3.49 ± 0.66	2.99 ± 0.65	**0.02**
Ultrasound muscle quality, no (%)			1
1: clearly visible	19 (95)	19 (95)	
2: still possible to spot muscle fascia and bone	1 (5)	1 (5)	
3: no evaluation possible	0 (0)	0 (0)	
Presence of oedema			0.47
1: no oedema	19 (95)	16 (80)	
2: slight indentation, rebound ≤ 15s	0 (0)	2 (10)	
3: deeper indentation, rebound 16-30s	1 (5)	1 (5)	
4: deeper indentation, rebound > 30s	0 (0)	0 (0)	
missing	0 (0)	1 (5)	
**Maximum handgrip strength (kgforce)**	39.4 ± 10.4	32.9 ± 8.9	**0.04**
**Functional Comorbidity Index (FCI), points, scale: 0-18**	0 (0-1)	1.5 (1-2)	**0.001**

**Clinical Frailty Scale on study day (CFS), points, scale: 1-9**	2 (1-2)	2 (2-3)	**<0.001**
**RAND-36 Physical Composite Score (PCS), points**	78.8 (77.5-81.4)	56.5 (50.3-72.1)	**<0.001**
**RAND-36 Mental Composite Score (MCS), points**	76.0 (72.6-78.2)	68.3 (55.8-75.3)	**0.01**

Table S6. Raw means and standard deviations for muscle protein fractional synthetic rate.

	Controls pre-intervention (n=20)	Controls post-intervention (n=20/19*)	ICU survivors pre-intervention (n=20)	ICU survivors post-intervention (n=20)
Fractional synthetic rate (%/hour), plasma precursor	0.0303±0.0085	0.0699±0.0119	0.0408±0.0109	0.0714±0.0164
Fractional synthetic rate (%/hour), muscle precursor	0.0566±0.0127	0.0933±0.0214	0.0662±0.0158	0.0923±0.0212

* For one control, post-intervention fractional synthetic rate could not be calculated from the intracellular/muscle amino acid precursor.

References

1. Lilja M, Mandić M, Apró W, Melin M, Olsson K, Rosenborg S, et al. High doses of anti-inflammatory drugs compromise muscle strength and hypertrophic adaptations to resistance training in young adults. Acta Physiol (Oxf). 2018;222(2).
2. Biolo G, Tipton KD, Klein S, Wolfe RR. An abundant supply of amino acids enhances the metabolic effect of exercise on muscle protein. Am J Physiol. 1997;273:E122–9.
3. Pennings B, Koopman R, Beelen M, Senden JM, Saris WH, van Loon LJ. Exercising before protein intake allows for greater use of dietary protein-derived amino acids for de novo muscle protein synthesis in both young and elderly men. Am J Clin Nutr. 2011;93:322–31.
4. Witard OC, Jackman SR, Breen L, Smith K, Selby A, Tipton KD. Myofibrillar muscle protein synthesis rates subsequent to a meal in response to increasing doses of whey protein at rest and after resistance exercise. Am J Clin Nutr. 2014;99:86–95.
5. Fischer A, Anwar M, Hertwig A, Hahn R, Pesta M, Timmermann I, Siebenrock T, Liebau K, Hiesmayr M. Ultrasound method of the USVALID study to measure subcutaneous adipose tissue and muscle thickness on the thigh and upper arm: an illustrated step-by-step guide. Clinical Nutrition Experimental. 2020;32:38–73.
6. Norrbrand L, Fluckey JD, Pozzo M, Tesch PA. Resistance training using eccentric overload induces early adaptations in skeletal muscle size. Eur J Appl Physiol. 2008;102(3):271–281.
7. Pozzo M, Alkner B, Norrbrand L, Farina D, Tesch PA. Muscle-fiber conduction velocity during concentric and eccentric actions on a flywheel exercise device. Muscle Nerve. 2006;34(2):169–177.
8. Rundqvist HC, Esbjörnsson M, Rooyackers O, Österlund T, Moberg M, Apro W, Blomstrand E, Jansson E. Influence of nutrient ingestion on amino acid transporters and protein synthesis in human skeletal muscle after sprint exercise. J Appl Physiol (1985). 2017 Dec 1;123(6):1501-1515.
