# Supplementary material for: Preserved anabolic skeletal muscle capacity in response to exercise and protein intake after critical illness
Source: Crit Care. 2026 Oct 24;30:549. doi: 10.1186/s13054-026-06418-7 (PMC13700533; doi:10.1186/s13054-026-06418-7)
Supplement: Supplementary file 2 [file 13054_2026_6418_MOESM2_ESM.pdf]

Study protocol 2021-04-26

Investigating the anabolic response to resistance exercise during and after critical illness.

Study acronym

ARTIST 1 & 2

Primary investigator

Martin Sundström Rehal, MD PhD

Version history

2022-02-15: Amendment to ethical review authority regarding screening and recruitment of patients in Study 2.
2022-03-10: Minor amendment regarding dose of oral protein and labeled phenylalanine in Study 2.
2022-07-10: Amendment to ethical review authority regarding exclusion criteria for therapeutic anticoagulation in Study 2.
2023-01-17: Minor amendment regarding exclusion criteria # 13 and 14 in Study 2.
2023-05-08: Amendment to ethical review authority regarding additional data collection (SF-36 and hand grip strength).
2023-10-23: Minor amendment regarding sample size target in Study 2.

Aim

The overall aim of this project is to determine the anabolic response to resistance exercise during and after critical illness.

Hypothesis

1) Resistance exercise, in addition to amino acid supplementation and routine physiotherapy, results in an improved muscle protein balance in ICU patients compared to amino acid supplementation and routine physiotherapy alone.

2) Patients recovering from critical illness have an impaired anabolic response to resistance exercise 3-6 months after ICU stay as compared to healthy controls.

Overview of the field

The debilitating impact of critical illness has been recognized for several decades. Disability related to intensive care is now described as a syndrome called ICU-acquired weakness (ICUAW). ICUAW affects up to 70% of ICU patients and is most common with higher illness severity. The pathophysiology is heterogenous, and involves muscle protein breakdown, altered bioenergetics, axonal injury and impaired excitation-contraction coupling. Patients that develop ICUAW require longer hospitalization and have a higher risk of death [1].

Weakness also has significant long-term consequences. In a landmark study, Herridge et al observed that predominantly young and previously healthy survivors of acute respiratory failure had reduced exercise capacity up to five years after ICU stay [2]. Physical function was unrelated to residual pulmonary dysfunction. Long-term disability was associated with significant health care costs, delayed return to work, and overall poor quality of life.

Preventing muscle atrophy is a potential way to counteract weakness. Critical illness is associated with a rapid loss of skeletal muscle, which may exceed 10% of lean body mass in the first week of intensive care [3,4]. Dos Santos et al found that a majority of ICU survivors had not recovered their muscle volume up to six months after hospital discharge [5].

During critical illness there is a dramatic increase in muscle protein breakdown, while synthesis is unaltered [6]. The result is a net loss of total body protein. Aging and comorbid conditions also contribute toward a resistance to anabolic signals, which may be exacerbated by immobilization and systemic inflammation [7]. Our research group has previously found that nutritional interventions such as amino acid supplementation can improve whole-body protein balance in ICU patients [8,9], but the effect on muscle metabolism is still unknown. Studies in exercise physiology have demonstrated that resistance training and amino acid ingestion have synergistic effects on muscle protein synthesis in healthy subjects [10]. It is therefore an appealing therapy to counteract muscle wasting in ICU, and to regain muscle mass during convalescence.

Despite several clinical trials, there remains equipoise regarding the efficacy of exercise in improving physical function in-ICU or after discharge [11,12]. These mixed signals are unsurprising given the heterogeneous causes of ICUAW. Only a few studies in this field assess

muscle architecture or cellular signaling in response to training [13,14]. However, the gold standard in determining the anabolic response to exercise is to directly measure the effect on protein synthesis and breakdown. To our knowledge there is still no published research using this methodology in critically ill patients.

Project description

The project consists of two studies. Study 1 will be conducted in the ICU. Study 2 will recruit study subjects who have been discharged from hospital after ICU stay.

Study populations:

Study 1 will recruit adult ICU patients suitable for active mobilization, which is already standard care in the unit at Karolinska University Hospital Huddinge.

Inclusion criteria:

1. Adult (≥18 years) ICU patient.
2. Suitable for active mobilization as determined by the attending physician and physiotherapist.
3. Not expected to be discharged or transferred from the unit within 24 h.
4. Functioning arterial line in situ.

Exclusion criteria:

1. Not able to provide informed consent.
2. Systemic anticoagulation with LMWH/UFH/DOAC in therapeutic dose range for deep vein thrombosis or pulmonary embolism, or dual antiplatelet therapy. If LMWH is administered twice daily, the patient is eligible for participation provided that vascular access is performed at nadir prior to the first daily dose.
3. Clinically significant inherited or acquired disorder of hemostasis.
4. Morbid obesity that interferes with femoral cannulation or doppler measurements.
5. Hemodynamic instability requiring ongoing volume resuscitation with crystalloid solutions or blood products.
6. Lower-limb amputee.
7. Lower-limb artheriosclerotic disease with critical ischemia.
8. Metastatic cancer or active hematological malignancy.
9. Inherited disorder of amino acid metabolism.
10. Chronic muscle, neuromuscular och neurologic disease with prior documentation of clinically significant lower-limb involvement.

11. Pregnancy.
12. Single organ failure not requiring invasive mechanical ventilation prior to enrollment.

Study 2 will include patients 3-6 months after hospital discharge. All patients treated for ≥3 days in an ICU at Karolinska University Hospital will be screened for eligibility.
Inclusion criteria:

1. Adult (≥18 years) subject previously admitted to an ICU at Karolinska University Hospital for ≥3 days and discharged from hospital (intervention group).

OR

2. Adult (≥18 years) subject without a history of ICU admission (control group).

Exclusion criteria (applies to both intervention and control group):

1. Not able to provide informed consent.
2. >6 months since ICU discharge.
3. Systemic anticoagulation with warfarin or dual antiplatelet therapy.
4. Clinically significant inherited or acquired disorder of hemostasis.
5. Lower-limb amputee.
6. Lower-limb artheriosclerotic disease with critical ischemia.
7. Recent fracture in lower limbs or significant osteoarthritis limiting movement in knee or hip joint.
8. Metastatic cancer or active hematological malignancy.
9. Inherited disorder of amino acid metabolism.
10. Chronic muscle, neuromuscular och neurologic disease with prior documentation of clinically significant lower-limb involvement.
11. Pregnancy.
12. Single organ failure not requiring invasive mechanical ventilation during ICU stay.
13. Intubated only for airway protection with no other organ failure(s) during ICU stay.
14. Planned postoperative care in ICU after elective cardiothoracic surgery.

Intervention and controls:

- Study 1: All patients will perform a physiotherapist-led session of standardized active mobilization. An intravenous infusion of mixed amino acids is administered concurrently.

Patients randomized to the intervention group will perform weighted resistance exercise with the leg catheterized for blood sampling as a part of their physiotherapy session.

- Study 2: All subjects will perform standardized lower extremity resistance exercise in conjunction with enteral protein supplementation. An age- and sex-matched group of healthy controls will be used for comparison.

Outcomes:

- Study 1: The primary outcome is the difference in change between the intervention and control groups in lower limb protein balance, from baseline to post-intervention. Other parameters of muscle protein kinetics (protein breakdown and synthesis) will be reported as secondary outcome measures.
- Study 2: The primary outcome is the difference between patients and healthy controls in fractional rate of muscle protein synthesis, from baseline to post-intervention. The impact of exercise on anabolic/catabolic signaling pathways in muscle fibres will be reported as secondary outcome measures. Planned post-hoc analyses include characterization of metabolomics and hormonal profiles in responders and non-responders.

Study protocols

Study 1

At the beginning of the protocol, a primed intravenous infusion of labeled ring-$^{2}H_{5}$-phenylalanine (bolus 2.94 μmol/kg, infusion rate 2.94 μmol/kg/h) and $^{2}H_{3}$-methylhistidine (bolus 0.06 μmol/kg, infusion rate 0.06 μmol/kg/h) is started to achieve a steady state of tracer dilution in arterial blood and continued until the end of the protocol. A femoral venous catheter is then sited under local anaesthesia for lower limb blood sampling, with the tip placed just above the junction of the greater saphenous vein and common femoral vein. The physician placing the catheter is free to determine the side of placement depending on the vascular anatomy on ultrasound investigation. A standardized measurement of upper and lower limb muscle thickness is also performed to quantify the baseline muscle mass of study subjects [15].

After 165 minutes of tracer infusion, four 2 ml samples from arterial and lower limb venous blood and femoral artery blood flow measurements on the side of the catheterized leg are performed at five minute intervals. Doppler blood flow measurements will be performed using a high-frequency linear probe with a fixed probe angulation at a level immediately cranial to the

branching of the deep femoral artery. The end-diastolic inner diameter of the vessel is determined at the same level. Immediately after the baseline measurements, an intravenous infusion of mixed amino acids (Glavamin, Fresenius Kabi) is administered at a maximum rate of 0.1 g/kg/h.

The study subjects will then participate in a physiotherapy session of protocolized active mobilization. While sitting on the side of the bed, patients in the intervention group will be encouraged to perform weighted leg extensions with the leg catheterized for blood sampling, targeting 8-12 repetitions to failure in three sets. The physiotherapist in charge will document the number of repetitions and other items of the mobilization protocol performed.

After returning to a supine or semi-recumbent position, new blood samples and blood flow measurements as described above are performed to determine the change in lower limb protein balance every 30 minutes up to 90 minutes post-intervention. At the completion of the protocol the infusions of labeled and unlabeled amino acids are stopped and the femoral vein catheter is removed. The protocol is illustrated in Figure 1.

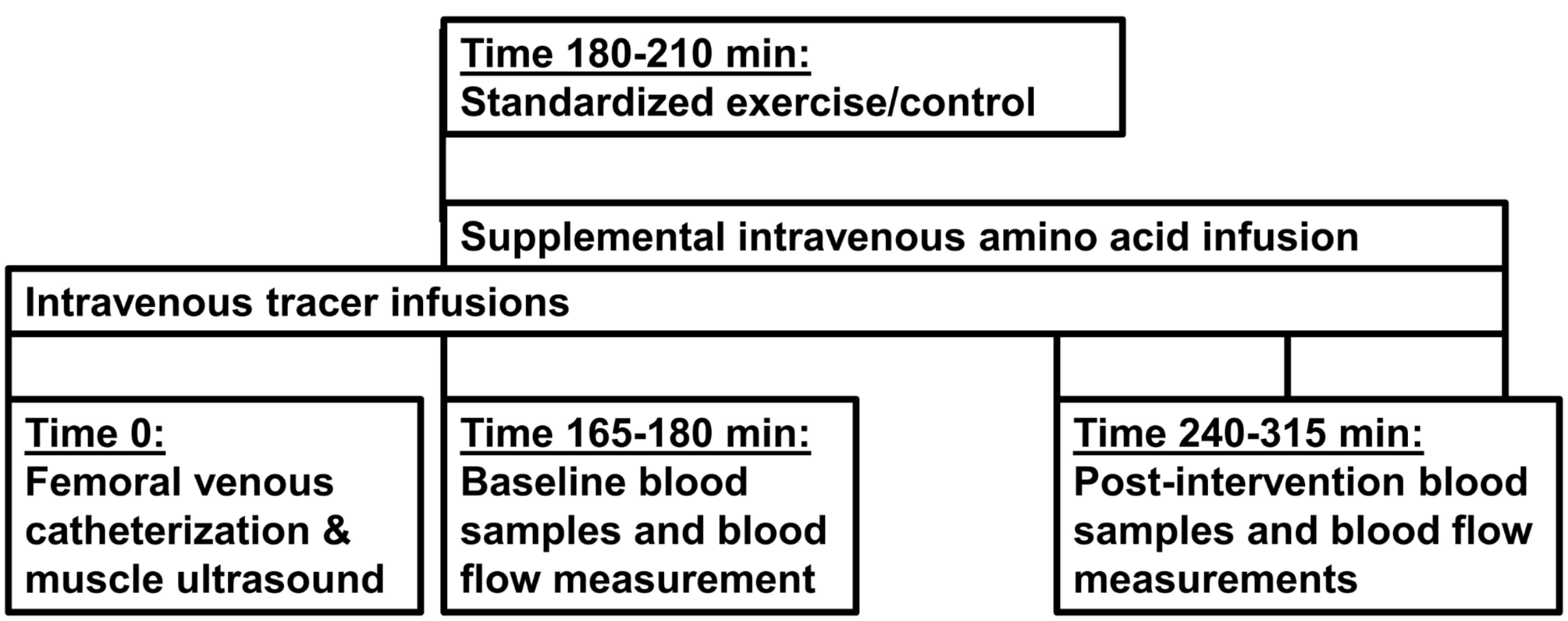

Figure 1. A schematic illustration of the protocol in Study 1 (not to scale).

All other aspects of treatment will be determined by the attending physician and care team, with a request that the rate of enteral and parenteral nutrition remains unchanged from 06:00 in the morning until the end of the protocol.

Study 2

Study subjects will arrive at the study site in the morning in a fasted state. After a baseline physical examination, a peripheral venous catheter and an arterial line are placed in an upper extremity under local anaesthesia. A primed intravenous infusion of ring-$^{2}H_{5}$-phenylalanine (bolus 2.94 µmol/kg, infusion rate 2.94 µmol/kg/h) is started to achieve steady state distribution of tracer between plasma and the intracellular precursor pool, and continued until the end of the protocol. During the infusion period a standardized ultrasound measurement of lower limb muscle thickness and grip strength is performed to quantify the baseline muscle mass and function of study subjects. Charlson Comorbidity Index, Clinical Frailty Scale and RAND 36-item Short Form Health Survey are reported through self-assessment. After 150 minutes of tracer infusion, a baseline muscle biopsy of ~150 mg is extracted from the non-dominant vastus lateralis muscle under local anaesthesia. Blood samples are drawn at baseline (plasma amino acids and lipid profile, inflammatory markers, white blood cell count, hormonal profile and metabolomics) and at 15 minute intervals during the infusion period to determine tracer enrichment.

Subjects will then perform a short program of standardized quadriceps resistance exercise with the dominant leg, consisting of leg extensions in a flywheel inertia training machine targeting ten repetitions in four sets. Afterwards the subject will ingest a commercially available protein supplement containing 24 g of hydrolyzed whey protein over 5 minutes. To avoid diluting the ratio of labeled/unlabeled phenylalanine, 235 µmol of ring-$^{2}H_{5}$-phenylalanine is added to the beverage. A second biopsy is extracted from the contralateral vastus lateralis muscle used in the training session 150 minutes from the start of protein intake. The protocol is illustrated in Figure 2.

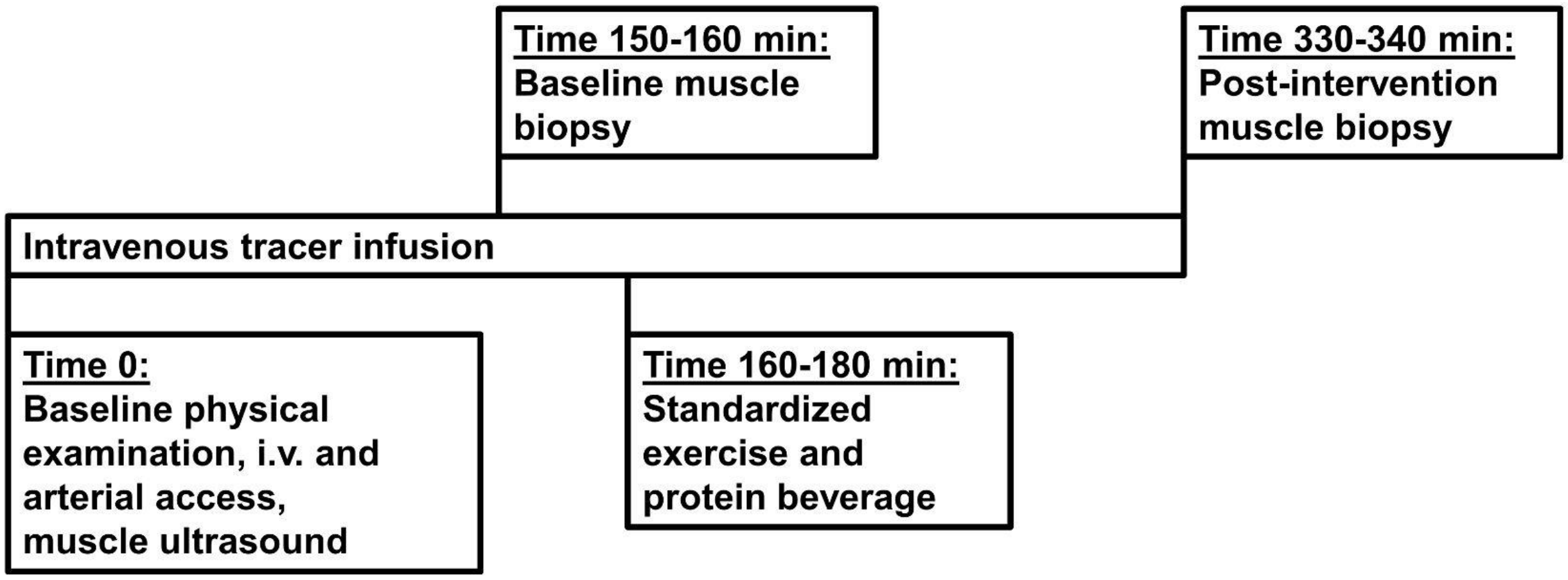

Figure 2. A schematic illustration of the protocol in Study 2 (not to scale).

Calculations

Study 1

Lower limb protein balance will be determined using a two-pool model [6]. Net balance (NB) of phenylalanine is derived using the Fick principle:

$NB = (C_A - C_V)*PF$

where C_A = arterial content (nmol/ml); C_V = venous content (nmol/ml); and PF = plasma flow (ml/min).

The rate of appearance (Ra) approximates protein breakdown and is calculated as:

$Ra = C_V*[1-(E_V/E_A)]*PF$

Where E_A and E_V are the arterial and venous enrichments of tracer expressed as molar percentage excess and calculated as an average of the four blood samples in each measurement series.

The rate of disappearance (Rd) approximates protein synthesis and is calculated as:

$Rd = NB + Ra$

Study 2

Fractional synthesis rate (FSR) of muscle protein is calculated as

$$FSR = (E_B(t_2) - E_B(t_1))/\int_{t1}^{t2} E_P(t)\ dt$$

Where E_B is the enrichment of bound amino acid, E_P enrichment of the precursor pool, t_1 and t_2 the duration of tracer infusion at the time of the first and second biopsy respectively.

Statistical considerations

Randomization

In Study 1, randomization will be determined using a computer-generated sequence in permuted blocks of four. Treatment allocation is concealed in opaque envelopes prepared by a research associate not involved in the study. The envelope is opened by the physiotherapist supervising the exercise session just before the intervention. Research staff in charge of outcome assessment are blinded to allocation until all data analysis is complete.

Sample size

As variation in muscle protein balance between ICU patients is very large [16], only paired comparisons will allow for studies of small-moderate size to find an effect. Unfortunately, there is no published data that describes the variation in repeated measures of muscle protein balance in ICU patients to support a formal power calculation. For Study 1, the best available estimate of effect size comes from pooled results on changes in whole-body protein balance during nutritional interventions. Using this data, we determine that 10 patients in each treatment arm are required to observe a mean change from negative to neutral lower limb muscle protein balance. To account for potential dropouts we will enroll a total of 24 patients.

For Study 2, there is no data from similar studies in this population on which to accurately estimate a treatment effect for sample size calculation. A target of 20 post-ICU patients and 20 matched controls has been chosen to allow for potential characterization of responders and non-responders in the post-intensive care group.

Statistics

Between-group differences of continuous outcomes will be analyzed using two-way repeated measures ANOVA. Within-group differences will be analyzed using one-way repeated measures ANOVA. A normal distribution of within-subject change in protein kinetics is assumed from previous observations. The predetermined level of significance is $p \leq 0.05$. Correction for multiple comparisons will not be applied and p-values for secondary outcomes should be considered as exploratory.

References

1. Batt J, Herridge MS, Dos Santos CC. From skeletal muscle weakness to functional outcomes following critical illness: a translational biology perspective. Thorax. Nov 2019;74(11):1091-1098. doi:10.1136/thoraxjnl-2016-208312
2. Herridge MS, Tansey CM, Matté A, et al. Functional disability 5 years after acute respiratory distress syndrome. N Engl J Med. Apr 2011;364(14):1293-304. doi:10.1056/NEJMoa1011802
3. Plank LD, Connolly AB, Hill GL. Sequential changes in the metabolic response in severely septic patients during the first 23 days after the onset of peritonitis. Ann Surg. Aug 1998;228(2):146-58. doi:10.1097/00000658-199808000-00002
4. Puthucheary ZA, Rawal J, McPhail M, et al. Acute skeletal muscle wasting in critical illness. JAMA. Oct 2013;310(15):1591-600. doi:10.1001/jama.2013.278481
5. Dos Santos C, Hussain SN, Mathur S, et al. Mechanisms of Chronic Muscle Wasting and Dysfunction after an Intensive Care Unit Stay. A Pilot Study. Am J Respir Crit Care Med. Oct 2016;194(7):821-830. doi:10.1164/rccm.201512-2344OC
6. Klaude M, Mori M, Tjäder I, Gustafsson T, Wernerman J, Rooyackers O. Protein metabolism and gene expression in skeletal muscle of critically ill patients with sepsis. Clin Sci (Lond). Feb 2012;122(3):133-42. doi:10.1042/CS20110233
7. Morton RW, Traylor DA, Weijs PJM, Phillips SM. Defining anabolic resistance: implications for delivery of clinical care nutrition. Curr Opin Crit Care. 2018 Apr;24(2):124-130. doi: 10.1097/MCC.0000000000000488.
8. Sundström Rehal M, Liebau F, Tjäder I, Norberg Å, Rooyackers O, Wernerman J. A supplemental intravenous amino acid infusion sustains a positive protein balance for 24 hours in critically ill patients. Crit Care. 2017 Dec 6;21(1):298. doi: 10.1186/s13054-017-1892-x.

9. Sundström Rehal M, Liebau F, Wernerman J, Rooyackers O. Whole-body protein kinetics in critically ill patients during 50 or 100% energy provision by enteral nutrition: A randomized cross-over study. PLoS One. 2020 Oct 5;15(10):e0240045. doi: 10.1371/journal.pone.0240045.
10. Wolfe RR. Skeletal muscle protein metabolism and resistance exercise. J Nutr. 2006 Feb;136(2):525S-528S. doi: 10.1093/jn/136.2.525S.
11. Doiron KA, Hoffmann TC, Beller EM. Early intervention (mobilization or active exercise) for critically ill adults in the intensive care unit. Cochrane Database Syst Rev. 2018 Mar 27;3(3):CD010754. doi: 10.1002/14651858.CD010754.pub2.
12. Connolly B, Salisbury L, O'Neill B, Geneen L, Douiri A, Grocott MP, Hart N, Walsh TS, Blackwood B; ERACIP Group. Exercise rehabilitation following intensive care unit discharge for recovery from critical illness. Cochrane Database Syst Rev. 2015 Jun 22;2015(6):CD008632. doi: 10.1002/14651858.CD008632.pub2.
13. Fossat G, Baudin F, Courtes L, Bobet S, Dupont A, Bretagnol A, Benzekri-Lefèvre D, Kamel T, Muller G, Bercault N, Barbier F, Runge I, Nay MA, Skarzynski M, Mathonnet A, Boulain T. Effect of In-Bed Leg Cycling and Electrical Stimulation of the Quadriceps on Global Muscle Strength in Critically Ill Adults: A Randomized Clinical Trial. JAMA. 2018 Jul 24;320(4):368-378. doi: 10.1001/jama.2018.9592.
14. Hickmann CE, Castanares-Zapatero D, Deldicque L, Van den Bergh P, Caty G, Robert A, Roeseler J, Francaux M, Laterre PF. Impact of Very Early Physical Therapy During Septic Shock on Skeletal Muscle: A Randomized Controlled Trial. Crit Care Med. 2018 Sep;46(9):1436-1443. doi: 10.1097/CCM.0000000000003263.
15. Fischer A, Anwar M, Hertwig A, Hahn R, Pesta M, Timmermann I, Siebenrock T, Liebau K, Hiesmayer M. Ultrasound method of the USVALID study to measure subcutaneous adipose tissue and muscle thickness on the thigh and upper arm: An illustrated step-by-step guide. Clinical Nutrition Experimental. 2020 Aug 1;32:38-73. doi: 10.1016/j.yclnex.2020.06.003
16. Gamrin-Gripenberg L, Sundström-Rehal M, Olsson D, Grip J, Wernerman J, Rooyackers O. An attenuated rate of leg muscle protein depletion and leg free amino acid efflux over time is seen in ICU long-stayers. Crit Care. 2018 Jan 23;22(1):13. doi: 10.1186/s13054-017-1932-6.
